# Nonparametric testing of lack of dependence in functional linear models

**DOI:** 10.1371/journal.pone.0234094

**Published:** 2020-06-26

**Authors:** Wenjuan Hu, Nan Lin, Baoxue Zhang

**Affiliations:** 1 School of Statistics, Capital University of Economics and Business, Beijing, China; 2 School of Mathematics and Statistics, Northeast Normal University, Changchun, Jilin Province, China; 3 Department of Mathematics, College of Arts and Sciences, Washington University in St. Louis, St. Louis, Missouri, United States of America; Cleveland Clinic Lerner Research Institute, UNITED STATES

## Abstract

An important inferential task in functional linear models is to test the dependence between the response and the functional predictor. The traditional testing theory was constructed based on the functional principle component analysis which requires estimating the covariance operator of the functional predictor. Due to the intrinsic high-dimensionality of functional data, the sample is often not large enough to allow accurate estimation of the covariance operator and hence causes the follow-up test underpowered. To avoid the expensive estimation of the covariance operator, we propose a nonparametric method called Functional Linear models with U-statistics TEsting (FLUTE) to test the dependence assumption. We show that the FLUTE test is more powerful than the current benchmark method (Kokoszka P,2008; Patilea V,2016) in the small or moderate sample case. We further prove the asymptotic normality of our test statistic under both the null hypothesis and a local alternative hypothesis. The merit of our method is demonstrated by both simulation studies and real examples.

## Introduction

Functional regression studies how a response variable *Y* varies with a functional predictor *X*(*s*), where *Y* can be scalar (Y∈R) or functional (*Y*(*t*) ∈ *L*^2^([0, 1])). The space *L*^2^([0, 1]) denotes the Hilbert space for square integrable functions. Without loss of generality, we define the index *s* and *t* on the closed interval [0, 1]. In case the raw support of *s* and *t* is a closed interval [*a*, *b*], one can simply rescale it to the interval [0, 1]. In this paper, we assume data following the widely used functional linear model (FLM) [1–4]. For a functional response, the FLM is defined as

$$\begin{array}{c} Y\left(t\right)=\alpha \left(t\right)+\int_{0}^{1}\beta \left(t,s\right)X\left(s\right)ds+\epsilon \left(t\right), \end{array}$$ (1)

where both the intercept *α*(*t*) and the random error *ϵ*(*t*) are square integrable and independent of *X*(*s*), the regression coefficient *β*(*t*, *s*) is in *L*^2^[0, 1] × *L*^2^[0, 1]. Denote that $B\left(X\right)=\int_{0}^{1}\beta \left(t,s\right)X\left(s\right)ds$, where B is the regression operator $B:L^{2}\left[0,1\right]\to L^{2}\left[0,1\right]$. For a scalar response variable *Y*, the FLM has a simpler form $Y=\alpha +\int_{0}^{1}X\left(s\right)\beta \left(s\right)ds+\epsilon$, where both the intercept *α* and the random error *ϵ* are real valued, and the regression parameter *β*(*s*) ∈ *L*^2^[0, 1]. Hereafter, we mainly focus on FLMs with functional responses, but the general methodology also applies to scalar responses.

In this paper, we consider testing whether the regression operator B has an assigned structure $B_{0}$, that is, to test

$$\begin{array}{c} H_{0}:\;\;B=B_{0}\;\;\text{versus}\;\;H_{1}:\;\;B\neq B_{0}. \end{array}$$ (2)

In practice, people often focus on the special case with $B_{0}=0$, i.e. to test the dependency between the response variable and the predictor. Existing tests in the literature for this problem can be categorized into parametric and nonparametric tests. In parametric tests, the test statistics are usually established by first estimating the functional regression coefficient through dimension reduction, such as functional PCA [5, 6–8]. Methods for real-valued responses include [6], [7] and [8]. [6] used a test statistic based on the *L*_2_ norm of the empirical cross-covariance operator of (*X*, *Y*). [8] proposed a Wald-type test with varying thresholds in selecting the number of principal components. [7] developed four test statistics based on the functional principal component (FPC) scores. They assume normality on the error distribution due to the need of the likelihood function. For functional responses, the test statistic proposed by [5] is constructed based on the eigenvalues and eigenfunctions decomposed from the functional PCA of the response variable *Y*(*t*) and the predictor *X*(*s*). Such parametric methods require the costly estimation of the covariance operator of the predictor. Due to the intrinsic high dimensionality of functional data, the inaccuracy and numerical instability in the covariance operator estimation may render the parametric tests invalid especially for small or moderate size samples. The same issue also occurs in high dimensional problems in multivariate statistics [9, 10]. Another limitation of FPC is that the principal component scores are computed independently from the predictor. Then the directions which explain *X*(*t*) best may not be the best predictors for the response which may lead to disparate test results for the regression problems. On the other hand, nonparametric tests utilized a different idea to avoid estimating the covariaznce operator [11, 12, 15]. For real-valued responses, [11] used the Nadaraya-Watson technique [13, 14] to estimate the conditional mean of $Y-B_{0}\left(X\right)$ given *X* = *x*. [15] also proposed a nonparametric test based on a kernel function for real responses. However, this method still requires estimating the covariance operator to calculate the semimetric. Furthermore, this test needs to split the sample into three groups, one of the three groups is used to estimate the kernel function, another to estimate the sample mean of responses variables, and the last group contributes to statistic, which is suitable for large sample data. The test proposed by [12] is for functional responses, and its test statistic is a weighted U-statistic with weights obtained from nearest neighbor smoothing. While this test possesses the correct Type-I error rate, identification of the neighbors requires defining distances between the functional predictors in the least favorable direction, which tends to result in lower power in general.

Motivated by [10], we propose a novel nonparametric test, called FLUTE, based on a U-statistic that measures the *L*_2_ distance in the induced space after transforming the original space of the functional predictor by the covariance operator. Our approach avoids explicit estimation of the covariance operator as it is based on the distance in the induced space. The FLUTE test can be applied to both real-valued and functional responses.

The paper is organized as follows. In Section Methodology, we introduce basic notations about functional linear regression model and the FLUTE test statistic. After presenting the theory in Section Asymptotic theory for functional responses, we further discuss the FLUTE test for FLMs with a scalar response in the next Section. Section Simulation and real data reports results from simulation studies and real data. The last section is the conclusion section.

## Methodology

### Notation and assumptions

Let 〈⋅, ⋅〉 denote the inner product in *L*^2^[0, 1], that is, for any *f*_1_, *f*_2_ ∈ *L*^2^[0, 1] $\langle f_{1},f_{2}\rangle =\int_{0}^{1}f_{1}\left(t\right)f_{2}\left(t\right)dt.$ The *L*_2_ norm ‖⋅‖ is defined by ‖*f*‖^2^ = 〈*f*, *f*〉. We assume in the FLM (1) that both the predictor *X*(*s*) and response variable *Y*(*t*) are random elements of *L*^2^[0, 1] and integrable. The sample functions *X_i_*(*s*), *i* = 1, …, *n*, are independently and identically distributed (i.i.d.) with *E*[*X*(*s*)] = *μ*_*x*_(*s*) and *E*‖*X*(*s*)‖^4^ < ∞. We also assume that the random trajectories *ϵ_i_*(*t*) are i.i.d with *E*[*ϵ*(*t*)] = 0, *E*[*ϵ*^2^(*t*)] = *σ*^2^(*t*) and *E*‖*ϵ*(*t*)‖^4^ < ∞.

Suppose {*ϕ*_*k*_, *k* ≥ 1} and {*η*_*ℓ*_, *ℓ* ≥ 1} are some orthonormal bases of the Hilbert space $H_{1}$ and $H_{2}$, respectively. To simplify the representation, hereafter we focus on the case where the Hilbert spaces $H_{1}$ and $H_{2}$ are *L*^2^[0, 1]. Then we represent the predictor *X_i_*(*s*) and the regression coefficient *β*(*t*, *s*) via the Karhunen-Loève decomposition [16]. For any *s* ∈ [0, 1], we have

$$\begin{array}{c} X_{i}\left(s\right)=\mu_{x}\left(s\right)+\sum_{k=1}^{\infty }\xi_{ik}\phi_{k}\left(s\right), \end{array}$$ (3)

where *μ*_*x*_(*s*) is the mean function of the predictor *X_i_*(*s*), and the expansion coefficient *ξ_ik_* = 〈*X_i_*, *ϕ_k_*〉, with E[*ξ_ik_*] = 0. For any *t*, *s* ∈ [0, 1], the regression coefficient *β*(*t*, *s*) is represented as

$$\begin{array}{c} \beta \left(t,s\right)=\sum_{k=1}^{\infty }\sum_{\ell =1}^{\infty }\beta_{\ell k}\eta_{\ell }\left(t\right)\phi_{k}\left(s\right), \end{array}$$ (4)

where *β*_*ℓk*_ = ∬*β*(*t*, *s*)*η*_*ℓ*_(*t*)*ϕ*_*k*_(*s*)*dtds*.

Next we introduce the covariance operator C of the predictor *X*(*s*) and its empirical counterpart $C_{n}$. For any element *f* ∈ *L*^2^([0, 1]), we define

$$\begin{array}{c} C\left(f\right)=E[\left\langle X-\mu_{x},f\right\rangle \left(X-\mu_{x}\right)]\;\text{and}\;C_{n}\left(f\right)=\frac{1}{n}\sum_{i=1}^{n}\left\langle X_{i}-\bar{X},f\right\rangle \left(X_{i}-\bar{X}\right), \end{array}$$

where $\bar{X}=\frac{1}{n}\sum_{i=1}^{n}X_{i}$. And denote the corresponding eigenelements by $C\left(\nu_{k}\right)=\lambda_{k}\nu_{k}$ with the eigenvalues λ_1_ ≥ λ_2_ ≥ … and *ν*_*k*_ the eigenfunction corresponding to λ_*k*_.

If both the predictor *X*(*s*) and response variable *Y*(*t*) are fully observed, hereafter we assume that *α*(*t*) = 0 and *μ*_*x*_(*s*) = 0 which will be explained in the next section, then the FLM with a functional response (1) can be represented as,

$$\begin{array}{c} Y\left(t\right)=\int_{0}^{1}\sum_{k=1}^{\infty }\sum_{\ell =1}^{\infty }\beta_{\ell k}\eta_{\ell }\left(t\right)\phi_{k}\left(s\right)\sum_{k^{\prime }=1}^{\infty }\xi_{k^{\prime }}\phi_{k^{\prime }}\left(s\right)ds+\epsilon \left(t\right). \end{array}$$ (5)

In practice, the infinite expansion (3), (4) and (5) above is usually approximated by a truncated basis expansion (e.g. B-spline basis and Fourier basis) [4, 11, 16, 17]. If the functional variables are densely observed, then recovering each trajectory of the functional variables based on the least square method is straightforward [18, 19]. If the functional variables are sparsely observed, [20] and [21] proposed to estimate the FPC scores through local linear surface smoother for the covariance operator, and then approximate each trajectory using the first *K* eigenfunctions. For the sparse observation, the error can not be ignored. Due to the certified complexity of the asymptotic normality of the statistic, we leave this area for future investigation.

We will represent the FLM with a functional response (1) using basis expansion when the approximation error is controlled. Denote *e_i_*(*t*) as the approximation error produced through $\sum_{k=1}^{K}\xi_{ik}\phi_{k}\left(s\right)$ and $\sum_{k=1}^{K}\sum_{\ell =1}^{L}\beta_{\ell k}\eta_{\ell }\left(t\right)\phi_{k}\left(s\right)$ approximating B(X), that is,

$$\begin{array}{c} e_{i}\left(t\right)=\int_{0}^{1}\beta \left(t,s\right)x_{i}\left(s\right)ds-\sum_{k=1}^{K}\sum_{\ell =1}^{L}\xi_{ik}\beta_{\ell k}\eta_{\ell }\left(t\right) \end{array}$$ (6)

Similar with Condition 1 in Appendix B in [22], then by the Cauchy-Schwarz inequality, uniformly across all *i* = 1, …, *n*, we have $\langle e_{i},e_{i}\rangle \leq 6\tilde{C}C/K^{4}+3C^{2}/L^{4}$ (Please see lemma 1), where *C* and $\tilde{C}$ are two positive constants.

As *K*, *L* → ∞, the approximation error should be more precise and become ignored. Hence the FLM with a functional response (1) can be rewritten as,

$$\begin{array}{c} Y_{i}\left(t\right)=\int_{0}^{1}\sum_{k=1}^{K}\sum_{\ell =1}^{L}\beta_{\ell k}\eta_{\ell }\left(t\right)\phi_{k}\left(s\right)\sum_{k=1}^{K}\xi_{ik}\phi_{k}\left(s\right)ds+\epsilon_{i}\left(t\right). \end{array}$$ (7)

In this paper, we assume that both the predictor *X*(*s*) and response variable *Y*(*t*) are fully observed or the approximation error is controlled.

### The FLUTE test

In this section, we introduce the FLUTE test whose test statistic is a U-statistic. The theory of U-statistics for fixed-dimensional data, pioneered by [23], has been well documented; see [24] and [25] for summaries. Recently, [10] developed the theory for high-dimensional multivariate data.

Under the functional linear model (1), if *α*(*t*) = 0 and E[*X*(*s*)] = 0, we can see that $\|Y\left(t\right)-B_{0}{\left(X\right)\|}^{2}=\langle B\left(X\right)-B_{0}\left(X\right)+\epsilon \left(t\right),B\left(X\right)-B_{0}\left(X\right)+\epsilon \left(t\right)\rangle$, which is then the perturbed *L*^2^ norm for measuring the distance between B(X) and $B_{0}\left(X\right)$. Further, it is easy to see that

$$\begin{array}{c} \begin{array}{cc} E[\|Y\left(t\right)-B_{0}{\left(X\right)\|}^{2}]= & \int \;\;\;\int \;\;\;\int \{\beta \left(t,s\right)-\beta_{0}\left(t,s\right)\}c\left(s,e\right)\{\beta \left(t,e\right)\\ & -\beta_{0}\left(t,e\right)\}dsdedt+\int \sigma^{2}\left(t\right)dt, \end{array} \end{array}$$ (8)

where *c*(*s*, *e*) = *E*[*X*(*s*)*X*(*e*)]. Note that $B=B_{0}$ is equivalent to the first term on the right-hand side of Eq (8) being zero. Thus we may consider testing the hypothesis (2) by a U-statistic with $\int (Y_{i}\left(t\right)-B_{0}\left(X_{i}\right))\langle X_{i},X_{j}\rangle (Y_{j}\left(t\right)-B_{0}\left(X_{j}\right))dt$ as the kernel, whose expectation is $\int \;\;\;\int C^{2}(\beta \left(t,s\right)-\beta_{0}\left(t,s\right))\left(t,e\right)dedt={\left\|C\left(\beta -\beta_{0}\right)\right\|}^{2}$, where C(β)=∫β(t,s)c(s,e)ds.

For the general case where *α*(*t*) ≠ 0 and *E*[*X*(*s*)] ≠ 0, we consider the U-statistic *T*_*n*_,

$$\begin{array}{c} T_{n}=\frac{1}{\left(\begin{array}{c} n\\ 4 \end{array}\right)}\sum_{A}\psi \left(i_{1},i_{2},i_{3},i_{4}\right), \end{array}$$ (9)

where $\psi \left(i_{1},i_{2},i_{3},i_{4}\right)=\frac{1}{3}\{\varphi \left(i_{1},i_{2},i_{3},i_{4}\right)+\varphi \left(i_{1},i_{3},i_{2},i_{4}\right)+\varphi \left(i_{1},i_{4},i_{2},i_{3}\right)\}$, and $\varphi \left(i_{1},i_{2},i_{3},i_{4}\right)=\frac{1}{4}\langle X_{i_{1}}-X_{i_{2}},X_{i_{3}}-X_{i_{4}}\rangle \int \pi_{i_{1},i_{2}}\pi_{i_{3},i_{4}}dt$, with $\pi_{i,j}=Y_{i}\left(t\right)-Y_{j}\left(t\right)-B_{0}\left(X_{i}-X_{j}\right)$, and $A=\{\left(i_{1},\ldots ,i_{4}\right):1\leq i_{1}<\ldots <i_{4}\leq n\}$ denotes combinations over all subscripts (*i*_1_, …, *i*_4_). As the statistic *T*_*n*_ is invariant to location shifts in both *X_i_*(*s*) and *Y_i_*(*t*), without loss of generality, we assume that *α*(*t*) = 0 and *μ*_*x*_(*s*) = 0 in the rest of the paper. Define *θ*(*F*) = *E*[*ψ*(*i*_1_, *i*_2_, *i*_3_, *i*_4_)], then *E*[*T*_*n*_] = *θ*(*F*).

As the statistic *T*_*n*_ measures the distance between the regression operator B and the assumed structure $B_{0}$ under the null hypothesis, large values of the statistic *T*_*n*_ are in favor of the alternative hypothesis and leads to rejection of the null hypothesis.

For the representation of the predictor *X*(*s*), we have

$$\begin{array}{c} X\left(s\right)=\xi^{\prime }\Phi \left(s\right), \end{array}$$ (10)

where Φ(*s*) = (*ϕ*_1_(*s*), …, *ϕ*_*K*_(*s*))′, *ξ* = (*ξ*_1_, …, *ξ_K_*)′, and *var*[ξ] = Σ. We next follow the general condition in [9] and assume that the loadings *ξ* of the predictor *X*(*s*) have a factor design structure.

**Assumption 1** There exists a *m*−variate random vector

**N** = (*N*_1_, …, *N*_*m*_)′ for some *m* < ∞ so that *ξ* = Γ*N*. Here Γ is a *K* × *m* matrix such that ΓΓ′ = Σ, and E[**N**] = **0**, var[**N**] = *I*_*m*_, where *I*_*m*_ is the *m* × *m* identity matrix. Each random variable *N*_*ℓ*_, *ℓ* = 1, …, *m*, is assumed to have finite 8th moment and $E(N_{\ell }^{4})=3+\rho$ for some constant *ρ* ∈ [0, 1). Further, for any $\sum_{d=1}^{D}\ell_{d}\leq 8$ and 1 ≤ *m*_1_ < *m*_2_ < … < *m*_*d*_ ≤ *m*, we assume $E\left[N_{m_{1}}^{\ell_{1}}N_{m_{2}}^{\ell_{2}}\ldots N_{m_{d}}^{\ell_{d}}\right]=E\left[N_{m_{1}}^{\ell_{1}}\right]E\left[N_{m_{2}}^{\ell_{2}}\right]\ldots E\left[N_{m_{d}}^{\ell_{d}}\right].$

Assumption 1 allows factors **N** to have a weak correlation. If the predictor *X*(*s*) follows a Gaussian process, [16] pointed out that *X*(*s*) admits the following expansion

$$\begin{array}{c} X\left(s\right)\overset{d}{=}\sum_{k=1}^{m}\sqrt{\lambda_{k}}N_{k}\nu_{k}\left(s\right), \end{array}$$

with independent standard normal random variables *N*_*k*_’s. It is easy to see that this is a special case of the factor design structure in Assumption 1, where the (*a*, *b*)*th* element of the transformation matrix Γ_*K*×*m*_ is $\sqrt{\lambda_{b}}\langle \nu_{b},\phi_{a}\rangle$.

Let *ε_i_* = (*ε*_*i*1_, …, *ε_iL_*)′ which is the expansion coefficients of *ϵ*(*t*), and Λ = var[ε]. We assume the following assumption.

**Assumption 2** For *i* ≠ *j*, $\text{E}\left[{\left(\varepsilon_{i}^{\prime }\varepsilon_{j}\right)}^{4}\right]=O\left({\text{tr}}^{2}\left(\Lambda^{2}\right)\right)$ and $\text{E}\left[{\left(\varepsilon_{i}^{\prime }\Lambda \varepsilon_{i}\right)}^{2}\right]=O\left({\text{tr}}^{2}\left(\Lambda^{2}\right)\right)$.

## Asymptotic theory

In this section, we derive the asymptotic unbiasedness of the FLUTE test and the asymptotic normality of its test statistic under both the null and a local alternative hypothesis through the Hoeffding decomposition.

Let *W_i_* = (*X_i_*(*s*), *ϵ_i_*(*t*)), where $\epsilon_{i}\left(t\right)=Y_{i}\left(t\right)-B_{0}\left(X_{i}\right)\left(t\right)$. Thus, *ψ*(*i*_1_, *i*_2_, *i*_3_, *i*_4_) in Eq (9) can be represented as $\psi (W_{i_{1}},W_{i_{2}},W_{i_{3}},W_{i_{4}})$. And *ψ*_*c*_(*w*_1_, …, *w*_*c*_) = E[*ψ*(*w*_1_, …, *w*_*c*_, *W*_*c* + 1_, …, *W*_4_)], be the projections of *ψ* to lower-dimensional sample spaces for *c* = 1, …, 4, where *w*_1_, …, *w*_*c*_ are fixed variables (e.g. $\psi_{1}\left(w_{i_{1}}\right)=\text{E}\left(\psi \left(w_{i_{1}},W_{i_{2}},W_{i_{3}},W_{i_{4}}\right)\right)$, $\psi_{2}\left(w_{i_{1}},w_{i_{2}}\right)=\text{E}\left(\psi \left(w_{i_{1}},w_{i_{2}},W_{i_{3}},W_{i_{4}}\right)\right)$, $\psi_{3}\left(w_{i_{1}},w_{i_{2}},w_{i_{3}}\right)=\text{E}\left(\psi \left(w_{i_{1}},w_{i_{2}},w_{i_{3}},W_{i_{4}}\right)\right)$). The specific forms have been given in the appendix of Proof of Theorem 1. Let *v*_*c*_ = var[*ψ*_*c*_] be the variance. Let ${\tilde{\psi }}_{c}=\psi_{c}-\theta \left(F\right)$, then we have the Hoeffding decompositions for *T*_*n*_ is $T_{n}-\theta \left(F\right)=\sum_{c=1}^{4}\left(\begin{array}{c} 4\\ c \end{array}\right){\left(\begin{array}{c} n\\ c \end{array}\right)}^{-1}V_{nc}$, where $V_{nc}=\sum_{A_{c}}g_{c}\left(w_{i_{1}},\ldots ,w_{i_{c}}\right)$ and $g_{c}\left(w_{1},\ldots ,w_{c}\right)={\tilde{\psi }}_{c}-\sum_{j=1}^{c-1}\sum_{A_{c}}g_{j}\left(w_{i_{1}},\ldots ,w_{i_{j}}\right)$ with $A_{c}=\left\{\left(i_{1},\ldots ,i_{c}\right):1\leq i_{1}<\ldots <i_{c}\leq n\right\}$. The decomposition for the variance of *T*_*n*_ is $\text{var}\left[T_{n}\right]={n4}^{-1}\sum_{c=1}^{4}\left(\begin{array}{c} 4\\ c \end{array}\right)\left(\begin{array}{c} n-4\\ 4-c \end{array}\right)v_{c}$. We assume that E[*ψ*^2^(*W*_1_, …, *W*_4_)] exists. The proofs of the Hoeffding decompositions can be found in [23] and also [24]. [10] recently showed that the decomposition also holds when the dimension of the predictor *K* increases to infinity. Based on Proposition 1 in [10], if we find the minimum *c*′ such that $v_{c}^{\prime }$, *c*′ = 1, 2, or 3, is of the same order as *v*_4_, then *T*_*n*_ will be dominated by the first *c*′ terms, so that

$$\begin{array}{c} T_{n}-\theta \left(F\right)=\sum_{c=1}^{c^{\prime }}\left(\begin{array}{c} c\prime \\ c \end{array}\right){\left(\begin{array}{c} n\\ c \end{array}\right)}^{-1}V_{nc}\left\{1+o_{p}\left(1\right)\right\}. \end{array}$$ (11)

**Theorem 1** *Under the FLM (* 1 *) and assuming Assumption (1)*,

*K*, *L* → ∞ *as n* → ∞, *we have*

(i) $E\left[T_{n}\right]={\left\|C\left(\beta -\beta_{0}\right)\right\|}^{2}$ *and* $var\left[T_{n}\right]=\left\{\frac{16}{n}v_{1}+\frac{72}{n(n-1)}v_{2}\right\}\left\{1+o\left(1\right)\right\};$
(ii) $T_{n}-{\left\|C\left(\beta -\beta_{0}\right)\right\|}^{2}=\left\{\frac{16}{n}V_{n1}+\frac{72}{n(n-1)}V_{n2}\right\}\left\{1+o_{p}\left(1\right)\right\},$

*where* $E\left[V_{n1}^{2}\right]=v_{1}$ *and* $E\left[V_{n2}^{2}\right]=v_{2}-2v_{1}.$

Please see the Proof of Theorem 1 in Appendix.

Let Δ = (*β*_*ℓk*_ − *β*_0,*ℓk*_)_*ℓ*,*k*_, where *β*_0,*ℓk*_ define the loadings of *β*_0_(*t*, *s*). And let *M_a_* = ΔΣ^*a*^Δ′, *a* = 0, 1, 2, 3 (e.g. Σ^0^ = *I*_*K*_, Σ^2^ = ΣΣ), *Q*_0_ = Γ′Γ, *Q*_1_ = Γ′Δ′ΔΓ, *Q*_2_ = Γ′ΣΔ′ΔΓ, *Q*_3_ = Γ′ΣΓ, *Q*_4_ = Γ′Δ′ΔΣΔ′ΔΓ. Under $H_{0}:B=B_{0}$, we have Δ = 0, and hence *Q*_1_ = *Q*_2_ = *Q*_3_ = *M_i_* = 0 for *i* = 0, 1, 2, 3. So it is obvious that *v*_1_ = 0, and *T*_*n*_ is then a degenerate U-statistic. Under this case, we have

$$\begin{array}{c} \text{var}\left[T_{n}\right]=\frac{2}{n(n-1)}\text{tr}\left(\Lambda^{2}\right)\text{tr}\left(\Sigma^{2}\right)\left\{1+o\left(1\right)\right\}. \end{array}$$

Next we show that the form of the variance for *T*_*n*_ also holds under a subclass of local alternative hypothesis $H_{1}$ specified by the following condition,

$$\begin{array}{c} \text{tr}\left(M_{1}\right)=o\left(1\right)\;\text{and}\;\text{tr}\left(M_{3}\right)=o\left\{n^{-1}\text{tr}\left(\Sigma^{2}\right)\right\}. \end{array}$$ (12)

Under the null hypothesis, the equation *v*_1_ = *o*(*n*^−1^ *v*_2_) holds with *v*_1_ = 0. Under the local hypothesis, the equation *v*_1_ = *o*(*n*^−1^ *v*_2_) still holds (see Appendix). The following theorem then states the asymptotic normality of our test statistic under this local alternative.

**Theorem 2**. *Under the FLM (* 1 *), assuming Assumptions (1) and (2), under either the null hypothesis* $H_{0}$ *or the local alternatives* $H_{1}$, *as n* → ∞, *we have*

$$\begin{array}{c} \frac{n}{\sqrt{2tr\left(\Lambda^{2}\right)tr\left(\Sigma^{2}\right)}}(T_{n}-\|C\left(\beta -\beta_{0}\right){\|}^{2})\overset{d}{\to }N\left(0,1\right). \end{array}$$

Please also see the Proof of Theorem 2 in Appendix.

For real data, the trace *tr*(Σ^2^) and *tr*(Λ^2^) need to be estimated. We use the estimator given in Chen and Qin [26], which was shown to be unbiased and ratio consistent, i.e. $\hat{\text{tr}(\Sigma^{2})}/\text{tr}\left(\Sigma^{2}\right)\overset{p}{\to }1$, under the null hypothesis or the local alternatives. Specifically, the estimator is given as

$$\begin{array}{c} \hat{\text{tr}(\Sigma^{2})}=R_{1n}-2R_{2n}+R_{3n}, \end{array}$$ (13)

where $R_{1n}=\frac{1}{K_{n}^{2}}\sum_{A}{\langle X_{i_{1}},X_{i_{2}}\rangle }^{2},$ $R_{2n}=\frac{1}{K_{n}^{3}}\sum_{A}\langle X_{i_{1}},X_{i_{2}}\rangle \langle X_{i_{2}},X_{i_{3}}\rangle$, and $R_{3n}=\frac{1}{K_{n}^{4}}\sum_{A}\langle X_{i_{1}},X_{i_{2}}\rangle \times \langle X_{i_{3}},X_{i_{4}}\rangle$ with $K_{n}^{m}=n!/\left(n-m\right)!$. Following the same idea, we can also construct a consistent estimator of tr(Λ^2^) under *H*_0_.

Following Theorem 2, the FLUTE test rejects $H_{0}$ at significant level *α* if

$$\begin{array}{c} nT_{n}\geq \sqrt{2\hat{\text{tr}(\Lambda^{2})}\hat{\text{tr}(\Sigma^{2})}}z_{\alpha }, \end{array}$$

where *z*_*α*_ is the upper *α*−quantile of *N*(0, 1).

Theorem 2 also implies that the asymptotic power of the proposed statistic under the local alternative is

$$\begin{array}{c} ϒ(\|\beta -\beta_{0}\left\|\right)=\Phi (-z_{\alpha }+\frac{n\|C\left(\beta -\beta_{0}\right){\|}^{2}}{\sqrt{2\text{tr}\left(\Lambda^{2}\right)\text{tr}\left(\Sigma^{2}\right)}}). \end{array}$$

The quantity $r_{n}\left(\beta -\beta_{0},\Sigma ,\Lambda \right)=n{\left\|C\left(\beta -\beta_{0}\right)\right\|}^{2}/\sqrt{2\text{tr}\left(\Lambda^{2}\right)\text{tr}\left(\Sigma^{2}\right)}$ can be viewed as a signal to noise ratio (SNR). If *r*_*n*_(*β* − *β*_0_, Σ, Λ) = *o*(1), it is obvious that the power converges to *α*. If *r*_*n*_(*β* − *β*_0_, Σ, Λ) is in the order of O(1), the power converges to 1.

## FLUTE for scalar responses

In the FLM with a scalar response,

$$\begin{array}{c} Y=\alpha +\int_{0}^{1}X\left(t\right)\beta \left(t\right)dt+\epsilon , \end{array}$$ (14)

where *Y* ∈ *R*. The null hypothesis for the scalar response is defined as

$$\begin{array}{c} H_{0}:\beta \left(t\right)=\beta_{0}\left(t\right)\;\text{versus}\;H_{1}:\beta \left(t\right)\neq \beta_{0}\left(t\right). \end{array}$$

The idea of the FLUTE method in Section Asymptotic theory directly applies and only requires slight modification toward the dimension of the response and functional regression coefficients. For example, the kernel of the FLUTE statistic ${\overset{ˇ}{T}}_{n}$ is (*Y_i_* − 〈*X_i_*, *β*_0_〉)〈*X_i_*(*s*), *X_j_*(*s*)〉(*Y_j_* − 〈*X_j_*, *β*_0_〉) with expectation $E\left[{\overset{ˇ}{T}}_{n}\right]=\overset{ˇ}{\theta }\left(F\right)={\left\|C\left(\beta -\beta_{0}\right)\right\|}^{2}$, where $C\left(\beta \right)=\int_{0}^{1}c\left(t,s\right)\beta \left(s\right)ds$. The expansion of parameter *β*(*t*) is $\beta \left(t\right)=\sum_{k=1}^{K}\beta_{k}\phi_{k}\left(t\right)$. The theory can be developed using the same idea as in Section Asymptotic theory. We distinguish by denoting the counterpart to notations in Section Asymptotic theory with a check mark. For example, the kernel of the FLUTE statistic for scalar response model (14) is denoted by ${\overset{ˇ}{\psi }}_{c}$, and its variance is ${\overset{ˇ}{\nu }}_{c}$. The following theorems show that the same asymptotic null distribution in Theorems 1 and 2 hold for the scalar response case.

**Theorem 3**. *Under the FLM with scalar response (* 14 *), assuming Assumption (1), when K* → ∞ *as n* → ∞, *we have*

(i) $E\left[{\overset{ˇ}{T}}_{n}\right]={\left\|C\left(\beta -\beta_{0}\right)\right\|}^{2}$ *and* $var\left[{\overset{ˇ}{T}}_{n}\right]=\left\{\frac{16}{n}{\overset{ˇ}{v}}_{1}+\frac{72}{n(n-1)}{\overset{ˇ}{v}}_{2}\right\}\left\{1+o\left(1\right)\right\},$
(ii) ${\overset{ˇ}{T}}_{n}-{\left\|C\left(\beta -\beta_{0}\right)\right\|}^{2}=\left\{\frac{16}{n}{\overset{ˇ}{V}}_{n1}+\frac{72}{n(n-1)}{\overset{ˇ}{V}}_{n2}\right\}\left\{1+o_{p}\left(1\right)\right\}.$

We consider the local alternative hypothesis as follows.

$$\begin{array}{c} \int \;\;\;\int \left(\beta \left(t\right)-\beta_{0}\left(t\right)\right)c\left(t,s\right)\left(\beta \left(s\right)-\beta_{0}\left(s\right)\right)dtds=o\left(1\right), \end{array}$$

and

$$\begin{array}{c} \int \;\;\;\int \left(\beta \left(t\right)-\beta_{0}\left(t\right)\right)c^{3}\left(t,s\right)\left(\beta \left(s\right)-\beta_{0}\left(s\right)\right)dtds=o\left\{n^{-1}tr\left(\Sigma^{2}\right)\right\}, \end{array}$$

where $c^{3}\left(t,s\right)=\sum_{i=1}^{\infty }\lambda_{i}^{3}\nu_{i}\left(t\right)\nu_{i}\left(s\right)$.

**Theorem 4**. *In the FLM with a scalar response (* 14 *), assume Assumption (1) and E*[*ϵ*^4^] *is finite*. *Under either the null hypothesis* $H_{0}$ *or the local alternatives* $H_{1}$, *as n* → ∞, *we have*

$$\begin{array}{c} \frac{n}{\sigma^{2}\sqrt{2tr(\Sigma^{2})}}({\overset{ˇ}{T}}_{n}-\|C\left(\beta -\beta_{0}\right){\|}^{2})\overset{d}{\to }N\left(0,1\right), \end{array}$$

*where σ*^2^ = *var*[*ϵ*].

The proofs of Theorems 3 and 4 are omitted because they can be proved in the same way as Theorems 1 and 2 except with slight modification to the notations to reflect the difference in dimensionality.

## Simulation and real data

In this section, we demonstrate the performance of the FLUTE method by simulation studies and an application to a real data example. For cases with functional responses, we compare the FLUTE method with the method in [1], which is constructed based on the functional PCA, we call KMSZ, and the nonparametric test in [12] is constructed by a weighted U-statistic and we name as NP. The KMSZ method depends on the functional principal components and is more suitable for large sample case which could estimate the covariance operator well. The test statistic of the NP method depends on so-called the least-favorable direction *γ* which is more suitable for the low dimension case. Under the simulation setup, this direction *γ* can be decided in three different ways: 1) Pre-estimate *γ* based on a super large simulated data set and then use it for all simulated data sets; 2) pre-estimate *γ* based on the data set generated at each level of |*β*|^2^ and then use it for simulated data sets generated at the same level of |*β*|^2^; and 3) estimate *γ* based on each simulated data set. The simulation results please see Table 1 and more details can be found in the Supplementary Material A in S1 File. Results reported in this section are based on the second way, which is consistent with applications of the NP method to real data.

**Table 1:** Size and power for NP test with different searching methods.

|*β*|^2^	case 1		case 2		case 3	
	*α* = 0.05	*α* = 0.1	*α* = 0.05	*α* = 0.1	*α* = 0.05	*α* = 0.1
0.00	0.059	0.100	0.059	0.100	0.020	0.021
0.02	0.062	0.117	0.082	0.128	0.038	0.039
0.04	0.088	0.140	0.393	0.497	0.042	0.042
0.06	0.090	0.151	0.604	0.708	0.119	0.119
0.08	0.115	0.185	0.755	0.831	0.062	0.062
0.10	0.150	0.227	0.858	0.905	0.185	0.185
0.20	0.246	0.359	0.989	0.994	0.731	0.731
0.30	0.268	0.384	0.998	0.999	0.204	0.204
0.40	0.347	0.467	0.999	0.999	0.922	0.922
0.50	0.359	0.463	0.995	0.998	0.988	0.988

For FLMs with a scalar response, neither the KMSZ nor NP method is applicable because the former involves functional PCA on the response and the latter requires computing the *L*^2^ norm between two functional response values. The nonparametric test proposed by [15] which we name as NETRF is for the scalar response. However, the NETRF test still requires estimating the covariance operator to calculate the semetric. Furthermore, this test needs sufficiently large sample data to provide accurate estimations of each group. Therefore, we do not directly compare DelSol’s method with our FLUTE test, but we conduct simulation studies for small/moderate sample cases to demonstrate the incapability of DelSol’s method under these scenarios. Here we choose the current comparison benchmark as the F-test proposed by [7]. [7] actually proposed four asymptotically equivalent tests which also depends on the functional components, and can be more suitable for large sample case. We use the F-test because it behaves the best of the four tests for small to moderate samples.

### Simulation results

We next conduct a simulation study to evaluate the empirical size and power of our FLUTE test for small to moderate samples with sample size *n* varying between 40 and 100. In each simulation, we generate 1,000 Monte Carlo samples. Our computer codes are written in R. For basis expansion and functional PCA, we use the implementation in the R package fda.

#### Functional response

**Fig 1:**
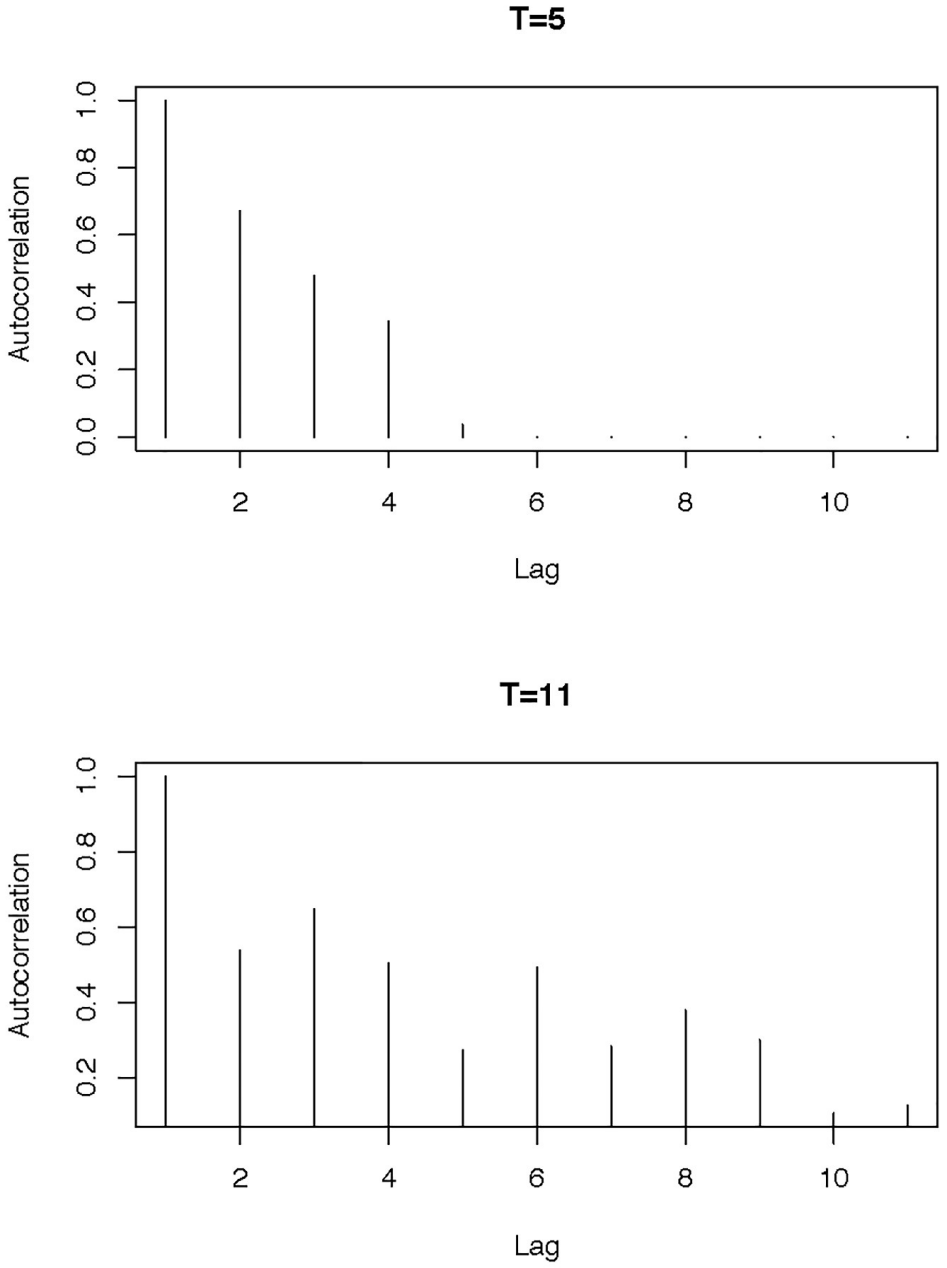
The autocorrelation functions for loadings ${\left\{\xi_{ik}\right\}}_{k=1}^{K}$.

First we present the case of the FLM with functional responses. This simulation design follows the FLM (1), where we set *β*(*t*, *s*) = |*β*|^2^ exp{(*t*^2^ + *s*^2^)/2}. Here |*β*|^2^ indicates the *L*_2_ norm of *β*(*t*, *s*) and is used to control the SNR. We generate the functional predictor *X*_*i*_(*s*) according to Eq (10), where the bases ${\left\{\phi_{k}\left(s\right)\right\}}_{k=1}^{K}$ are chosen as Fourier bases. For instance, the first five orthonormal Fourier basis functions are $\phi_{1}\left(s\right)=1,\phi_{2}\left(s\right)=\sqrt{2}\text{sin}\left(2\pi s\right)$, $\phi_{3}\left(s\right)=\sqrt{2}\text{cos}\left(2\pi s\right)$, $\phi_{4}\left(s\right)=\sqrt{2}\text{sin}\left(4\pi s\right)$, and $\phi_{5}\left(s\right)=\sqrt{2}\text{cos}\left(4\pi s\right)$. Without loss of generality, we set the mean *μ*_*x*_(*s*) = 0. According to the factor design (Assumption 1), the loadings *ξ*_1_, …, *ξ_n_* are independently generated from the following moving average model,

$$\begin{array}{c} \xi_{ik}=\rho_{1}N_{ik}+\rho_{2}N_{i(k+1)}+\ldots +\rho_{T}N_{i(k+T-1)},\;k=1,\ldots ,K, \end{array}$$ (15)

where the constant *T* controls range of dependency(see Fig 1). The coefficients ${\left\{\rho_{t}\right\}}_{t=1}^{T}$ are randomly generated from *U*(0, 1), where *U*(*a*, *b*) denotes the Uniform distribution on the interval (*a*, *b*). And the random vectors **N** *_i_* = (*N*_*i*1_, …, *N*_*i*(*K*+*T*−1)_)′ are independently generated from the *N*(0, *I*_*K* + *T*−1_) distribution. It then follows that the (*k*, *ℓ*)th element of the covariance matrix var(*ξ*) is $\sum_{t=1}^{T-|k-\ell |}\rho_{t}\rho_{t+|k-\ell |}I\{|k-\ell |<T\}$, which shows that the correlation between *ξ_ik_* and *ξ_iℓ_* is controlled by |*k* − *ℓ*| and *T*. The random error function *ε*_*i*_(*t*) is generated according to the decomposition in Eq (10). We also set bases ${\left\{\eta_{\ell }\left(t\right)\right\}}_{\ell =1}^{L}$ in the same way as {*ϕ*_*k*_(*s*)}. And the loadings *ε*_*i*1_, …, *ε_iL_* are independent identical distribution and generate from *N*(0, Σ_*ϵ*_).

To evaluate the impact of dimensionality and sample size, we carry out simulations under four different settings, varying in dimensionality and sample size *K* = *L* = 5 (low-dimensional) and *K* = *L* = 11 (high-dimensional), *n* = 40 and 100. When generating *X*(*t*), we set *T* = 5 in Eq (15). The variances of the loadings *ε*_*i*1_, …, *ε_iL_* are the same, we set Σ_*ϵ*_ = *I*_*L*_. Under each setting, we vary the |*β*|^2^ at 10 levels from 0 to 0.5 (see Tables 2 and 3). When |*β*|^2^ is 0, the result provides the empirical size of all tests, and results at the other 9 levels give the power. Each testing method is evaluated at two nominal significance levels *α* = 0.05 and 0.1.

**Table 2:** Size and power for different tests at *α* = 0.05.

|*β*|^2^	*K* = *L* = 5, *n* = 40			*K* = *L* = 11, *n* = 40			*K* = *L* = 5, *n* = 100			*K* = *L* = 11, *n* = 100		
	NP	KMSZ	FLUTE	NP	KMSZ	FLUTE	NP	KMSZ	FLUTE	NP	KMSZ	FLUTE
0.00	0.043	0.042	**0.072**	0.043	0.025	**0.055**	0.048	0.051	**0.065**	0.035	0.037	**0.057**
0.02	0.096	0.178	**0.482**	0.040	0.107	**0.445**	0.408	0.699	**0.848**	0.104	0.317	**0.620**
0.04	0.132	0.361	**0.799**	0.034	0.174	**0.781**	0.788	0.973	**0.997**	0.217	0.762	**0.873**
0.06	0.198	0.600	**0.932**	0.046	0.256	**0.907**	0.939	0.990	**1.000**	0.335	0.896	**0.969**
0.08	0.267	0.711	**0.973**	0.041	0.341	**0.968**	0.986	0.999	**1.000**	0.543	0.954	**0.998**
0.1	0.300	0.801	**0.992**	0.034	0.380	**0.979**	0.995	1.000	**1.000**	0.747	0.996	**1.000**
0.2	0.514	0.980	**1.000**	0.033	0.619	**0.998**	1.000	1.000	**1.000**	0.853	0.999	**1.000**
0.3	0.667	1.000	**1.000**	0.048	0.707	**0.999**	1.000	1.000	**1.000**	0.910	1.000	**1.000**
0.4	0.751	0.998	**1.000**	0.045	0.745	**1.000**	1.000	1.000	**1.000**	0.995	1.000	**1.000**
0.5	0.789	1.000	**1.000**	0.040	0.772	**1.000**	1.000	1.000	**1.000**	1.000	1.000	**1.000**

**Table 3:** Size and power for different tests at *α* = 0.1.

|*β*|^2^	*K* = *L* = 5, *n* = 40			*K* = *L* = 11, *n* = 40			*K* = *L* = 5, *n* = 100			*K* = *L* = 11, *n* = 100		
	NP	KMSZ	FLUTE	NP	KMSZ	FLUTE	NP	KMSZ	FLUTE	NP	KMSZ	FLUTE
0.00	0.079	0.093	**0.128**	0.080	0.068	**0.111**	0.077	0.085	**0.103**	0.078	0.079	**0.097**
0.02	0.165	0.322	**0.557**	0.084	0.208	**0.536**	0.520	0.803	**0.896**	0.126	0.342	**0.665**
0.04	0.193	0.535	**0.851**	0.067	0.326	**0.836**	0.862	0.990	**0.999**	0.248	0.784	**0.889**
0.06	0.288	0.735	**0.948**	0.082	0.460	**0.942**	0.964	0.996	**1.000**	0.326	0.899	**0.983**
0.08	0.353	0.843	**0.980**	0.073	0.525	**0.984**	0.991	1.000	**1.000**	0.533	0.905	**0.999**
0.1	0.392	0.909	**0.996**	0.066	0.605	**0.988**	0.998	1.000	**1.000**	0.738	0.998	**1.000**
0.2	0.635	0.997	**1.000**	0.062	0.805	**1.000**	1.000	1.000	**1.000**	0.866	1.000	**1.000**
0.3	0.768	1.000	**1.000**	0.085	0.885	**0.999**	1.000	1.000	**1.000**	0.930	1.000	**1.000**
0.4	0.821	1.000	**1.000**	0.076	0.903	**1.000**	1.000	1.000	**1.000**	0.997	1.000	**1.000**
0.5	0.860	1.000	**1.000**	0.074	0.911	**1.000**	1.000	1.000	**1.000**	1.000	1.000	**1.000**

Table 2 shows the empirical sizes and power obtained for different dimensionality and sample size under the nominal significance level *α* = 0.05. Under the same sample size, the power of all three tests decrease as the dimensions *K* and *L* increase. When the dimensionality is the same, the power of all three methods improves as the sample size increases. Table 2 also shows that the FLUTE method performs stably in both the low dimensional cases and the high dimensional cases. The KMSZ and NP tests are conservative and their power decreases significantly as the dimension increases.

Further the NP method has almost no power in the case of high dimension and small sample size (*K* = *L* = 11, *n* = 40). It is apparent that in Table 2, power of the FLUTE method is consistently higher than that of the KMSZ and NP methods, especially in high dimensional cases. The simulation results also show that the FLUTE method respects the nominal levels under high dimensionality at both sample size *n* = 40 and 100.

Table 3 shows the results under the nominal significance level *α* = 0.1, and provides the same conclusion as Table 2.

To evaluate the impact of the correlation structure, we carry out simulations under two different settings, *T* = 5 (weakly correlated) and 11 (strongly correlated) (see Fig 1). Under each setting, we vary |*β*|^2^ at 6 levels from 0 to 0.5 (see Table 4). When *T* is 5, the correlation is weak. Table 4 shows the empirical sizes and power obtained for the case of *K* = *L* = 11 and *n* = 40. The FLUTE method is stable for different *T*, both of the KMSZ and the NP method are more sensitive to the correlation structure. On the other hand, the power of the NP statistic decreases significantly when T increases, since this method needs to search the least-favorable direction. While the power of the KMSZ statistic decreases significantly when T reduces, since this method depends on functional PCA. When the correlation is weak, the number of functional PCs would increase to achieve the same percentage of variance explanation, hence the number of p also increase which results in lower power.

**Table 4:** Size and power for different correlation when *K* = *L* = 11, *n* = 40.

|*β*|^2^	*T* = 5, *α* = 0.05			*T* = 11, *α* = 0.05			*T* = 5, *α* = 0.1			*T* = 11, *α* = 0.1		
	NP	KMSZ	FLUTE	NP	KMSZ	FLUTE	NP	KMSZ	FLUTE	NP	KMSZ	FLUTE
0.00	0.050	0.041	**0.064**	0.043	0.034	**0.066**	0.086	0.082	**0.098**	0.086	0.100	**0.107**
0.10	0.580	0.452	**0.940**	0.238	0.849	**1.000**	0.675	0.652	**0.959**	0.348	0.954	**1.000**
0.20	0.794	0.740	**0.999**	0.340	0.946	**1.000**	0.824	0.894	**0.999**	0.443	0.991	**1.000**
0.30	0.860	0.863	**1.000**	0.404	0.971	**1.000**	0.884	0.959	**1.000**	0.523	0.998	**1.000**
0.40	0.883	0.883	**1.000**	0.433	0.983	**1.000**	0.903	0.975	**1.000**	0.537	0.998	**1.000**
0.50	0.900	0.929	**1.000**	0.443	0.982	**1.000**	0.911	0.986	**1.000**	0.562	0.998	**1.000**

To evaluate the performance of the FLUTE method with heteroscedastic variance, we carry out simulations under the following settings, the designed variances of the expansion coefficients of *ε_i_*(*t*) are Var(*ε_iℓ_*) = 1/*ℓ*, for *i* = 1, …, *n*, *ℓ* = 1, …, *L*. We set *T* = 5, *n* = 40. And we vary |*β*|^2^ at 6 levels from 0 to 0.5. The significance levels are *α* = 0.05 and *α* = 0.1 respectively. Table 5 shows the empirical sizes and power for the cases of *K* = *L* = 5 and *K* = *L* = 11, and provides a similar conclusion as Tables 2 and 3 when *n* = 40. The power of all three tests decreases as the dimensions *K* and *L* increase. However, the FULTE method performs stably in both low dimensional cases and the high dimensional cases, the power of the KMSZ and NP tests decreases significantly as the dimension increases.

**Table 5:** Size and power for heteroscedastic variance when *K* = *L* = 11, *n* = 40.

|*β*|^2^	*K* = *L* = 5, *α* = 0.05			*K* = *L* = 5, *α* = 0.1			*K* = *L* = 11, *α* = 0.05			*K* = *L* = 11, *α* = 0.1		
	NP	KMSZ	FLUTE	NP	KMSZ	FLUTE	NP	KMSZ	FLUTE	NP	KMSZ	FLUTE
0.00	0.055	0.042	**0.061**	0.088	0.102	**0.095**	0.057	0.033	**0.055**	0.092	0.092	**0.087**
0.10	0.972	1.000	**1.000**	0.981	1.000	**1.000**	0.116	0.404	**0.771**	0.188	0.628	**0.873**
0.20	0.998	1.000	**1.000**	0.999	1.000	**1.000**	0.176	0.647	**0.972**	0.286	0.813	**0.986**
0.30	0.999	1.000	**1.000**	1.000	1.000	**1.000**	0.308	0.793	**0.993**	0.427	0.918	**0.997**
0.40	1.000	1.000	**1.000**	1.000	1.000	**1.000**	0.408	0.808	**0.997**	0.518	0.929	**0.998**
0.50	0.998	1.000	**1.000**	0.999	1.000	**1.000**	0.474	0.829	**1.000**	0.589	0.951	**1.000**

**Fig 2:**
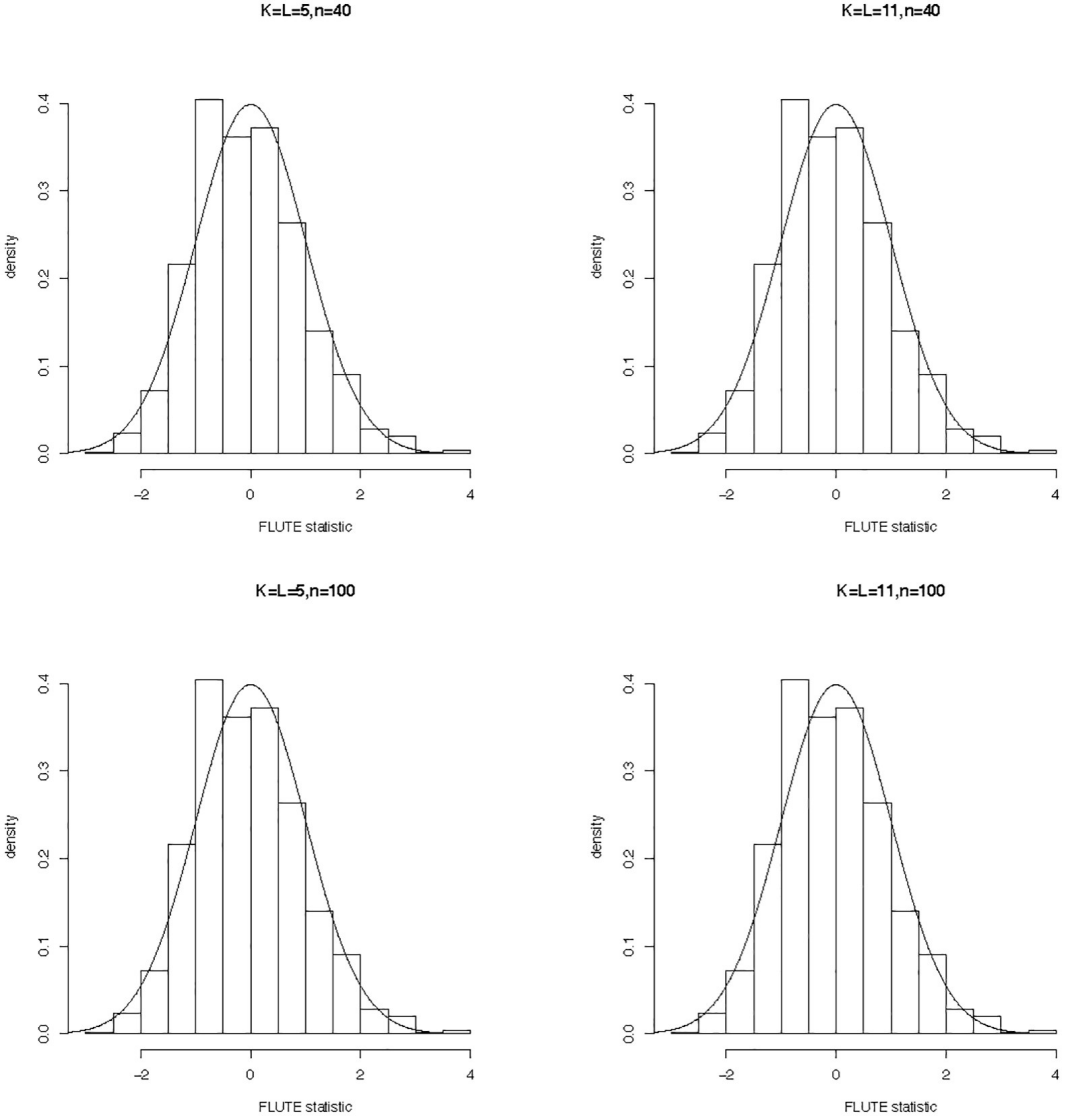
The null distribution of the FLUTE statistic in FLMs with functional responses. The solid line indicates the density of the standard normal distribution.

Fig 2 shows the histograms of the FLUTE statistic for different dimensionality and sample size under the null hypothesis, which matches nicely with the imposed standard normal density. This is consistent with our results in Theorm 2.

**Fig 3:**
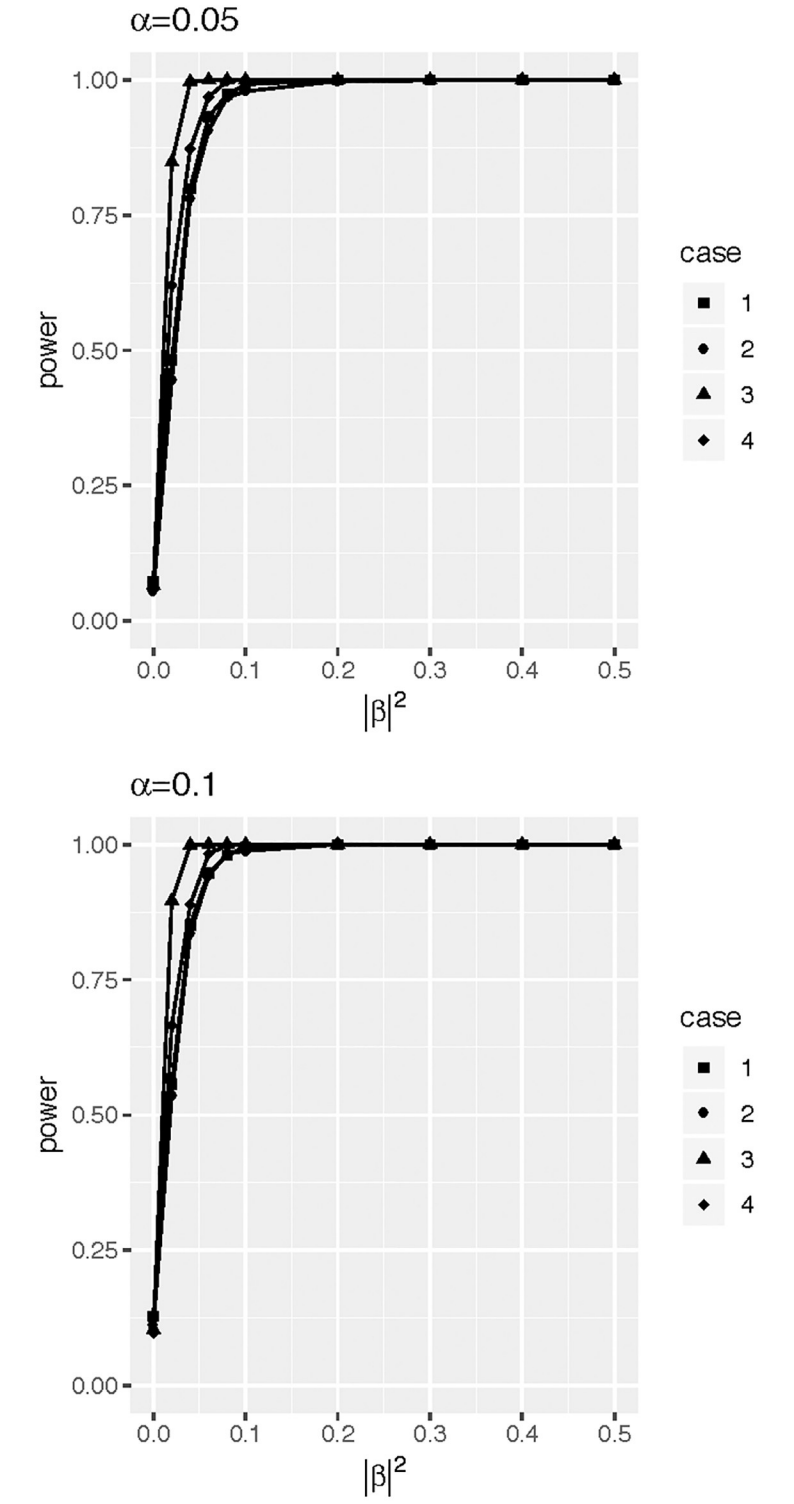
Power curves of the FLUTE method. Case 1: *K* = *L* = 5 and *n* = 40; Case 2: *K* = *L* = 11 and *n* = 40; Case 3: *K* = *L* = 5 and *n* = 100; Case 4: *K* = *L* = 11 and *n* = 100. The left figure is for *α* = 0.05, and the right is for *α* = 0.1.

Fig 3 shows the power curves of the FLUTE statistic under four different cases with varying dimensionality and sample size when the nominal significance level *α* = 0.05 and level *α* = 0.1. Under all the four cases, power curves have effective size, and when |*β*|^2^ is 0.2, the four power curves almost reached 1.

#### Scalar response

This section presents the results for FLMs with scalar responses. This simulation design follows the model (14). We set the coefficient of regression parameter as $\beta_{k}=\frac{1}{\sqrt{K}}{\left|\beta \right|}^{2},k=1,2,\ldots ,K$. The functional predictor *X*(*t*) is generated in the same way as in Section Functional response. And the random errors *ε_i_* are independently generated from *N*(0, 1).

Same with FLMs with functional responses in Section Functional response, we carry out simulations under four different settings, *K* = 5 (low-dimensional) and *K* = 11 (high-dimensional), *n* = 40 and 100. Under each setting, we vary |*β*|^2^ at 10 levels from 0 to 0.5 (see Tables 5 and 6). Each testing method is evaluated at two nominal significance levels *α* = 0.05 and 0.1.

**Table 6:** Size and power for normal residual at significant level *α* = 0.05.

|*β*|^2^	*K* = 5, *n* = 40		*K* = 11, *n* = 40		*K* = 5, *n* = 100		*K* = 11, *n* = 100	
	F-test	FLUTE	F-test	FLUTE	F-test	FLUTE	F-test	FLUTE
0.00	0.046	**0.047**	0.044	**0.040**	0.048	**0.055**	0.043	**0.057**
0.02	0.271	**0.379**	0.267	**0.403**	0.603	**0.771**	0.584	**0.791**
0.04	0.480	**0.664**	0.452	**0.680**	0.934	**0.968**	0.924	**0.973**
0.06	0.676	**0.822**	0.671	**0.820**	0.979	**0.993**	0.982	**0.991**
0.08	0.778	**0.882**	0.760	**0.891**	0.995	**0.999**	0.997	**0.999**
0.10	0.848	**0.931**	0.881	**0.957**	0.998	**1.000**	1.000	**1.000**
0.20	0.980	**0.995**	0.989	**0.995**	1.000	**1.000**	1.000	**1.000**
0.30	0.999	**1.000**	1.000	**1.000**	1.000	**1.000**	1.000	**1.000**
0.40	0.999	**1.000**	1.000	**1.000**	1.000	**1.000**	1.000	**1.000**
0.50	1.000	**1.000**	1.000	**1.000**	1.000	**1.000**	1.000	**1.000**

Table 6 shows the empirical sizes and powers obtained for different dimensionality and sample size under the nominal significance level *α* = 0.05. The power of the two tests, FLUTE and the F-test in [7], is similar in these four cases. The results show that the FLUTE test is more powerful than the F-test. Table 7 shows the same conclusions at nominal significance level *α* = 0.1.

**Table 7:** Size and power for normal residual at significant level *α* = 0.1.

|*β*|^2^	*K* = 5, *n* = 40		*K* = 11, *n* = 40		*K* = 5, *n* = 100		*K* = 11, *n* = 100	
	F-test	FLUTE	F-test	FLUTE	F-test	FLUTE	F-test	FLUTE
0.00	0.097	**0.098**	0.098	**0.104**	0.104	**0.104**	0.091	**0.092**
0.02	0.375	**0.449**	0.381	**0.472**	0.718	**0.818**	0.728	**0.829**
0.04	0.632	**0.712**	0.584	**0.739**	0.962	**0.975**	0.953	**0.982**
0.06	0.782	**0.857**	0.769	**0.858**	0.990	**0.997**	0.953	**0.982**
0.08	0.854	**0.912**	0.851	**0.914**	0.998	**1.000**	0.990	**0.995**
0.10	0.905	**0.947**	0.948	**0.974**	1.000	**1.000**	0.999	**0.999**
0.20	0.992	**0.995**	0.997	**0.996**	1.000	**1.000**	1.000	**1.000**
0.30	0.999	**1.000**	1.000	**1.000**	1.000	**1.000**	1.000	**1.000**
0.40	0.999	**1.000**	1.000	**1.000**	1.000	**1.000**	1.000	**1.000**
0.50	1.000	**1.000**	1.000	**1.000**	1.000	**1.000**	1.000	**1.000**

Table 8 shows the comparison between FLUTE and Delsol’s method at significant level *α* = 0.1, and *K* = 11. NETRF1, NETRF2 and NETRF3 stand for three bootstrap methods in Delsol’s paper. Due to the three test statistics are nonparametric tests that are constructed based on a kernel function, the estimation of bias and variance terms seems difficult. Further, it is usually irrelevant to use the quantiles of the asymptotic law to estimate the threshold directly. Thus, the bootstrap procedure is needed. For all three methods, we choose the semi-metric induced by functional principal components, and split the samples into three groups as 20, 10 and 10, when *n* = 40, 40, 30 and 30, when *n* = 100. Under each setting, the empirical significance level are calculated by 1000 bootstrap iterations. FLUTE stands for our method. It is obviously that the sizes of Delsol’s methods can not be well controlled at the nominal level for small/moderate samples.

**Table 8:** Size for normal residual at significant level *α* = 0.1.

*Y* = *ε*	NETRF1	NETRF2	NETRF3	FLUTE
n = 40	0.138	0.142	0.146	0.099
n = 100	0.121	0.127	0.123	0.096

### Application to Canadian Weather data

*The Canadian Weather data* is available from the R package fda (http://www.r-project.org) which named CanadianWeather. The data consists of the daily temperature and rainfall registered in 35 weather stations in Canada averaged over 1960 to 1994, hence the sample size is 35. We view the daily temperature as the predictor and the rainfall as the response variable. Both the predictor and the response variable are functional. We use the FLUTE test to check the dependency between the daily temperature and the rainfall. Following [3], we choose 11 Fourier bases to fit the temperature curve and rainfall curve for each station separately.

**Fig 4:**
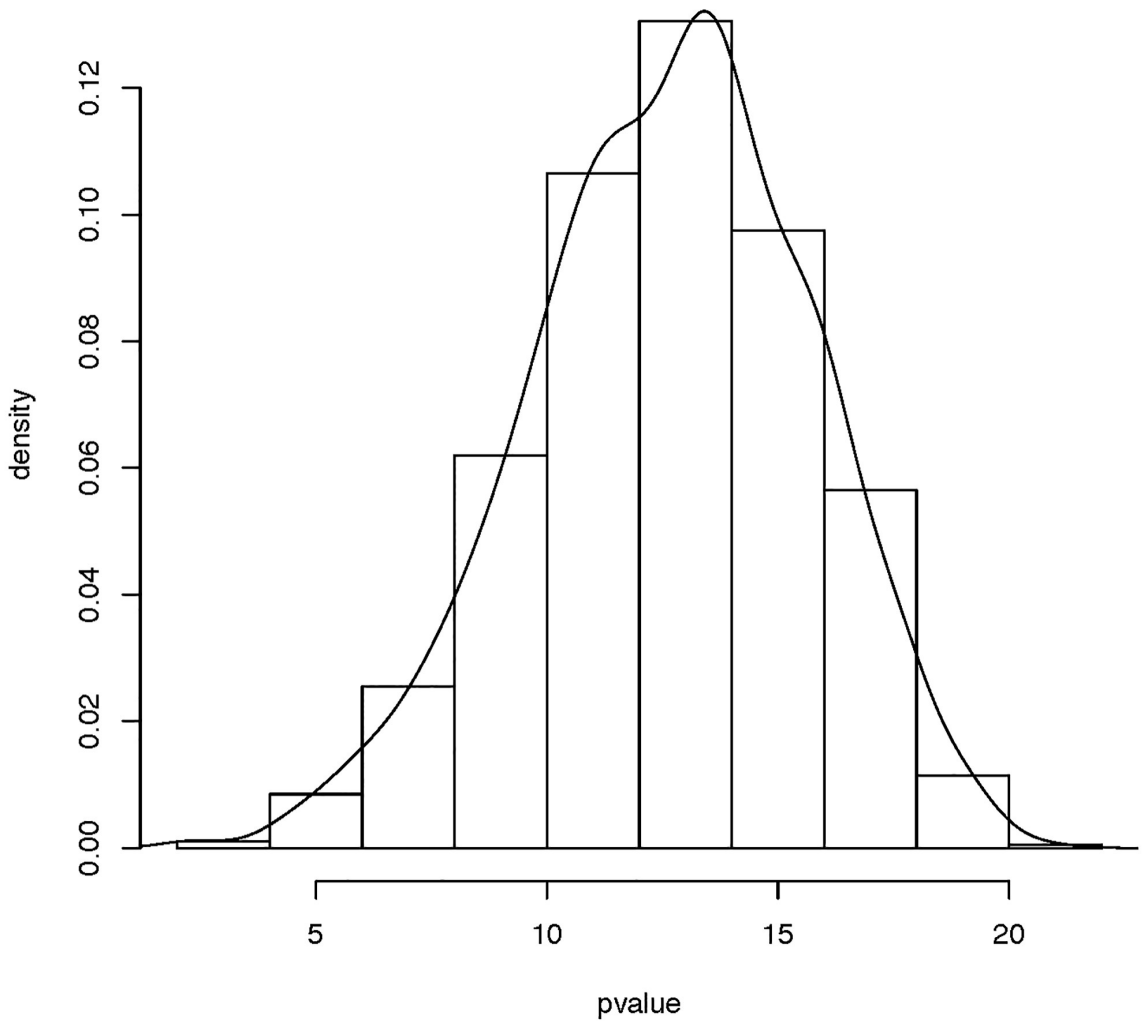
Empirical distribution of the FLUTE statistic based on 1000 bootstrap samples of size 35 drawn from the Canadian Weather dataset.

Let *Y_i_*(*t*) represent the logarithm of the rainfall at the station *i* at time t and *x_i_*(*t*) be the temperature of the same station at time *t* of the year. The value of FLUTE statistic is 12.17159 based on the whole 35 stations, hence we reject the null hypothesis. To illustrate the efficacy of the test, we repeat the test on 1000 bootstrap samples. Each bootstrap sample consists of data at 35 randomly selected stations with replacement from the total 35 stations. Fig 4 shows that the density of the FLUTE statistic is far away from the standard normal distribution, hence we prefer to reject the null hypothesis.

## Conclusion

We proposed the FLUTE test for testing dependence between the response and functional predictor in FLMs with either a real or functional response. By constructing a U-statistic that measures the *L*_2_ distance in an induced space, the FLUTE statistic avoids estimating the covariance operator of the predictor. The parametric test in [1] requires estimation of the covariance operator and demands large samples. The nonparametric test in [12], although avoids explicitly estimating the covariance operator, requires estimating the least-favorable direction *γ*. In general, using the least-favorable direction leads to lower power. Meanwhile, our experience suggests the estimation of *γ* can be numerically unstable across different simulated data sets, which results in poor test performance.

Our FLUTE test does not suffer from these problems. It requires minimum effort in estimating model parameters, hence achieves higher power, especially for high dimensional cases. One potential weakness of the FLUTE test is its high computational cost in evaluating a U-statistic in large samples. However, estimating covariance operator is less a concern in large samples, one can switch to using parametric methods. We recommend the best context of using the FLUTE test is small to moderate sample problems.

### Appendix

**Proof of Theorems**.

**Lemma 1**. *Suppose the functional predictors* {*X_i_*, *i* = 1, …, *n*} *and the regression function β*(*t*, *s*) *satisfy the following two conditions*,

(A) *Functional predictors*, {*X_i_*, *i* = 1, …, *n*}, *belongs to a Sobolev ellipsoid of order two: there exists a universal constant C, such that* $\sum_{k=1}^{\infty }\xi_{ik}^{2}k^{4}\leq C^{2}$ for all *i* = 1, …, *n*.
(B) *The regression functions satisfy* $\iint \beta^{2}\left(t,s\right)dtds\leq \tilde{C}$ *with some constant* $\tilde{C}$. *Further as L* → ∞, *the summation of coefficients* $\sum_{\ell =L+1}^{\infty }\beta_{k,\ell }\leq 1/L^{4}$ *for k* = 1, 2, ….

*then we have the approximation error* $\langle e_{i},e_{i}\rangle \leq 6\tilde{C}C/K^{4}+3C^{2}/L^{4}$.

*Proof*. Recall that

$$\begin{array}{ccc} e_{i}\left(t\right) & = & \int_{0}^{1}\beta \left(t,s\right)x_{i}\left(s\right)ds-\sum_{k=1}^{K}\sum_{\ell =1}^{L}\xi_{ik}\beta_{\ell k}\eta_{\ell }\left(t\right)\\ & = & \sum_{\ell =1}^{L}\sum_{k=K+1}^{\infty }\xi_{k}\beta_{\ell k}\eta_{\ell }\left(t\right)+\sum_{\ell =L+1}^{\infty }\sum_{k=1}^{K}\xi_{k}\beta_{\ell k}\eta_{\ell }\left(t\right)+\sum_{\ell =L+1}^{\infty }\sum_{k=K+1}^{\infty }\xi_{k}\beta_{\ell k}\eta_{\ell }\left(t\right)\\ & \overset{def}{=} & A_{1}+A_{2}+A_{3}. \end{array}$$

Then by the Cauchy-Schwarz inequality, we have

$$\begin{array}{c} \left\langle e_{i},e_{i}\right\rangle =\left\langle A_{1}+A_{2}+A_{3},A_{1}+A_{2}+A_{3}\right\rangle \leq 3\left(\left\langle A_{1},A_{1}\right\rangle +\left\langle A_{2},A_{2}\right\rangle +\left\langle A_{3},A_{3}\right\rangle \right). \end{array}$$

Next we show the three parts are controlled separately. According to the Holder inequality and Condition (A), we have

$$\begin{array}{ccc} \left\langle A_{1},A_{1}\right\rangle & = & \left\langle \sum_{\ell =1}^{L}\sum_{k=K+1}^{\infty }\xi_{ik}\beta_{\ell k}\eta_{\ell }\left(t\right),\sum_{\ell =1}^{L}\sum_{k=K+1}^{\infty }\xi_{ik}\beta_{\ell k}\eta_{\ell }\left(t\right)\right\rangle \\ & = & \sum_{k,k^{\prime }=K+1}^{\infty }\sum_{\ell =1}^{L}\xi_{ik}\xi_{ik^{\prime }}\beta_{\ell k}\beta_{\ell k^{\prime }}\\ & \leq & \sum_{k,k^{\prime }=K+1}^{\infty }\xi_{ik}\xi_{ik}^{\prime }{\left(\sum_{\ell =1}^{L}\beta_{\ell k}^{2}\sum_{\ell^{\prime }=1}^{L}\beta_{\ell^{\prime }k^{\prime }}^{2}\right)}^{\frac{1}{2}}\\ & = & {\left(\sum_{k=K+1}^{\infty }\xi_{ik}{\left(\sum_{\ell =1}^{L}\beta_{\ell k}^{2}\right)}^{\frac{1}{2}}\right)}^{2}\\ & \leq & \sum_{k=K+1}^{\infty }\xi_{ik}^{2}k^{4}\sum_{k=K+1}^{\infty }\sum_{\ell =1}^{L}\beta_{\ell k}^{2}k^{-4}\\ & \leq & \frac{C^{2}\tilde{C}}{K^{4}}. \end{array}$$ (16)

And we have

$$\begin{array}{ccc} \left\langle A_{2},A_{2}\right\rangle & = & \left\langle \sum_{\ell =L+1}^{\infty }\sum_{k=1}^{K}\xi_{ik}\beta_{\ell k}\eta_{\ell }\left(t\right),\sum_{\ell =L+1}^{\infty }\sum_{k=1}^{K}\xi_{ik}\beta_{\ell k}\eta_{\ell }\left(t\right)\right\rangle \\ & = & \sum_{k,k^{\prime }=1}^{K}\sum_{\ell =L+1}^{\infty }\xi_{ik}\xi_{ik^{\prime }}\beta_{\ell k}\beta_{\ell k^{\prime }}\\ & \leq & \sum_{k,k^{\prime }=1}^{K}\xi_{ik}\xi_{ik^{\prime }}{\left(\sum_{\ell =L+1}^{\infty }\beta_{\ell k}^{2}\sum_{\ell^{\prime }=L+1}^{\infty }\beta_{\ell^{\prime }k^{\prime }}^{2}\right)}^{\frac{1}{2}}\\ & = & {\left(\sum_{k=1}^{K}\xi_{ik}{\left(\sum_{\ell =L+1}^{\infty }\beta_{\ell k}^{2}\right)}^{\frac{1}{2}}\right)}^{2}\\ & \leq & \frac{1}{L^{4}}\sum_{k=1}^{K}\xi_{ik}^{2}k^{4}\sum_{k=1}^{K}k^{-4}\\ & \leq & \frac{C^{2}}{L^{4}}. \end{array}$$ (17)

Similar with the proof of Eqs (16) and (17), we get

$$\begin{array}{ccc} \left\langle A_{3},A_{3}\right\rangle & = & \left\langle \sum_{\ell =L+1}^{\infty }\sum_{k=K+1}^{\infty }\xi_{ik}\beta_{\ell k}\eta_{\ell }\left(t\right),\sum_{\ell =L+1}^{\infty }\sum_{k=K+1}^{\infty }\xi_{ik}\beta_{\ell k}\eta_{\ell }\left(t\right)\right\rangle \\ & \leq & \frac{C^{2}\tilde{C}}{K^{4}}. \end{array}$$ (18)

Hence we complete the proof by combining the bounds on each of the three parts.

Next, to prove Theorems 1 and 2, we first introduce some lemmas.

**Lemma 2**. *Suppose random vector* $Z_{i}={\left(z_{i1},\ldots ,z_{ip}\right)}^{\prime }\in R^{p},i=1,2$, *satisfy* *E*[*Z_i_*] = 0, *var*[*Z_i_*] = *I_p_*, $\text{E}[Z_{ik}^{4}]=3+\rho ,k=1,\ldots ,p$, *where ρ is a constant in* (0, 1). *If the two random variables Z*_1_ *and Z*_2_ *are independent, for any square matrix M* = (*m*_*kℓ*_)_*p*×*p*_, *we have*

(1) $E\left[Z_{1}Z_{1}^{{}^{\prime }}MZ_{1}Z_{1}^{{}^{\prime }}\right]=M+M^{\prime }+\text{tr}\left(M\right)I_{p}+\rho diag\left(M\right)$;
(2) $E\left[Z_{1}Z_{2}^{{}^{\prime }}MZ_{2}Z_{1}^{{}^{\prime }}\right]=\text{tr}\left(M\right)I_{p};$
(3) $E\left[Z_{1}Z_{2}^{{}^{\prime }}MZ_{1}Z_{2}^{{}^{\prime }}\right]=M^{\prime }.$

*Proof*.

(1) Let $W_{1}=Z_{1}Z_{1}^{{}^{\prime }}MZ_{1}Z_{1}^{{}^{\prime }}$, where *W*_1_(*k*, *ℓ*) indicates the (*k*, *ℓ*)*th* element of *W*_1_. With direct computation, we have *W*_1_(*k*, *ℓ*) = *Z*_1*k*_ *Z*_1*ℓ*_∑_*i*,*j*_ *m_ij_Z*_1*i*_ *Z*_1*j*_. If *k* = *ℓ*,
  $$\begin{array}{c} \text{E}\left[W_{1}\left(k,\ell \right)\right]=m_{kk}\text{E}\left[Z_{1k}^{4}\right]+\sum_{i\neq k}m_{ii}\text{E}\left[Z_{k}^{2}\right]\text{E}\left[Z_{i}^{2}\right]=\sum_{i}m_{ii}+\left(2+\rho \right)m_{kk}; \end{array}$$
  If *k* ≠ *ℓ*, E[*W*_1_(*k*, *ℓ*)] = *m*_*kℓ*_ + *m*_*ℓk*_. Then E[*W*_1_] = *M* + *M*′+ tr(*M*)*I*_*p*_ + *ρdiag*(*M*).
(2) Since $\text{E}\left[Z_{1}Z_{2}^{{}^{\prime }}MZ_{2}Z_{1}^{{}^{\prime }}\right]=\text{E}\left[Z_{1}Z_{1}^{{}^{\prime }}\right]\text{E}\left[Z_{2}^{{}^{\prime }}MZ_{2}\right]$, and $\text{E}\left[Z_{2}^{{}^{\prime }}MZ_{2}\right]=\sum_{i}m_{ii}$, then we have $\text{E}\left[Z_{1}Z_{2}^{{}^{\prime }}MZ_{2}Z_{1}^{{}^{\prime }}\right]=\text{tr}\left(M\right)I_{p}.$
(3) It’s simple to show that $\text{E}\left[Z_{1}Z_{2}^{{}^{\prime }}MZ_{1}Z_{2}^{{}^{\prime }}\right]=\text{E}\left[Z_{1}Z_{1}^{{}^{\prime }}\right]M^{\prime }\text{E}\left[Z_{2}Z_{2}^{{}^{\prime }}\right]=M^{\prime }$.

**Lemma 3**. *Consider symmetrical and semi-positive definite matrices A and B*, [27] *has improved some inequalities*:

(1) *tr*(*AB*)^2^ ≤ *tr*(*A*^2^)*tr*(*B*^2^);
(2) *tr*^2^(*AB*) ≤ *tr*(*A*^2^)*tr*(*B*^2^).

**Lemma 4**. *For matries M*_*a*_, *a* = 1, 2, 3 *defined as M*_*a*_ = ΔΣ^*a*^Δ′, *we have tr*^2^(*M*_2_) ≤ *tr*(*M*_1_)*tr*(*M*_3_).

*Proof*.

$$\begin{array}{c} \text{tr}\left(M_{2}\right)=\text{tr}\left(\Delta \Sigma^{\frac{1}{2}}\cdot \Sigma^{\frac{3}{2}}\Delta^{{}^{\prime }}\right)\leq \sqrt{\text{tr}\left(\Delta \Sigma^{\frac{1}{2}}\Sigma^{\frac{1}{2}}\Delta^{{}^{\prime }}\right)\text{tr}\left(\Delta \Sigma^{\frac{3}{2}}\Sigma^{\frac{3}{2}}\Delta^{{}^{\prime }}\right)}=\sqrt{\text{tr}\left(M_{1}\right)\text{tr}\left(M_{3}\right)}. \end{array}$$

**Proof of Theorem** 1.

Recall that the definition of the statistic in Eq (9), it is straightforward to show that $E\left[T_{n}\right]={\left\|C\left(\beta -\beta_{0}\right)\right\|}^{2}$.

To find the dominating terms, we need to calculate the following projections,

$$\begin{array}{c} \psi_{1}\left(w_{1}\right)=\frac{1}{2}\{\text{tr}(\Delta \left(\xi_{1}\xi_{1}^{{}^{\prime }}+\Sigma \right)\Sigma \Delta^{{}^{\prime }})+\text{tr}\left(\varepsilon_{1}\xi_{1}^{{}^{\prime }}\Sigma \Delta^{{}^{\prime }}\right)\}, \end{array}$$

$$\begin{array}{c} \begin{array}{cc} & \psi_{2}\left(w_{1},w_{2}\right)\\ & =\frac{1}{6}\{\text{tr}(\Delta \left(\xi_{1}-\xi_{2}\right){\left(\xi_{1}-\xi_{2}\right)}^{{}^{\prime }}\Sigma \Delta^{{}^{\prime }})+\text{tr}(\left(\varepsilon_{1}-\varepsilon_{2}\right){\left(\xi_{1}-\xi_{2}\right)}^{{}^{\prime }}\Sigma \Delta^{{}^{\prime }})\\ & +\text{tr}(\Delta \left(\xi_{1}\xi_{1}^{{}^{\prime }}+\Sigma \right)\left(\xi_{2}\xi_{2}^{{}^{\prime }}+\Sigma \right)\Delta^{{}^{\prime }})+\text{tr}(\varepsilon_{1}\xi_{1}^{{}^{\prime }}\left(\xi_{2}\xi_{2}^{{}^{\prime }}+\Sigma \right)\Delta^{{}^{\prime }})\\ & +\text{tr}(\Delta \left(\xi_{1}\xi_{1}^{{}^{\prime }}+\Sigma \right)\xi_{2}\varepsilon_{2}^{{}^{\prime }})+\text{tr}(\varepsilon_{1}\xi_{1}^{{}^{\prime }}\xi_{2}\varepsilon_{2}^{{}^{\prime }})\}, \end{array} \end{array}$$

$$\begin{array}{c} \begin{array}{cc} & \psi_{3}\left(w_{1},w_{2},w_{3}\right)\\ & =\frac{1}{12}\{\text{tr}(\Delta \left(\xi_{1}-\xi_{2}\right){\left(\xi_{1}-\xi_{2}\right)}^{{}^{\prime }}\left(\xi_{3}\xi_{3}^{{}^{\prime }}+\Sigma \right)\Delta^{{}^{\prime }})\\ & +\text{tr}(\left(\varepsilon_{1}-\varepsilon_{2}\right){\left(\xi_{1}-\xi_{2}\right)}^{{}^{\prime }}\left(\xi_{3}\xi_{3}^{{}^{\prime }}+\Sigma \right)\Delta^{{}^{\prime }})+\;\text{tr}(\Delta \left(\xi_{1}-\xi_{2}\right){\left(\xi_{1}-\xi_{2}\right)}^{{}^{\prime }}\xi_{3}\varepsilon_{3}^{{}^{\prime }})\\ & +\;\text{tr}(\left(\varepsilon_{1}-\varepsilon_{2}\right){\left(\xi_{1}-\xi_{2}\right)}^{{}^{\prime }}\xi_{3}\varepsilon_{3}^{{}^{\prime }})+\text{tr}(\Delta \left(\xi_{1}-\xi_{3}\right){\left(\xi_{1}-\xi_{3}\right)}^{{}^{\prime }}\left(\xi_{2}\xi_{2}^{{}^{\prime }}+\Sigma \right)\Delta^{{}^{\prime }})\\ & +\text{tr}(\left(\varepsilon_{1}-\varepsilon_{3}\right){\left(\xi_{1}-\xi_{3}\right)}^{{}^{\prime }}\left(\xi_{2}\xi_{2}^{{}^{\prime }}+\Sigma \right)\Delta^{{}^{\prime }})+\text{tr}(\Delta \left(\xi_{1}-\xi_{3}\right){\left(\xi_{1}-\xi_{3}\right)}^{{}^{\prime }}\xi_{2}\varepsilon_{2}^{{}^{\prime }})\\ & +\text{tr}(\left(\varepsilon_{1}-\varepsilon_{3}\right){\left(\xi_{1}-\xi_{3}\right)}^{{}^{\prime }}\xi_{2}\varepsilon_{2}^{{}^{\prime }})+\text{tr}(\Delta \left(\xi_{1}\xi_{1}^{{}^{\prime }}+\Sigma \right)\left(\xi_{2}-\xi_{3}\right){\left(\xi_{2}-\xi_{3}\right)}^{{}^{\prime }}\Delta^{{}^{\prime }})\\ & +\text{tr}(\varepsilon_{1}\xi_{1}^{{}^{\prime }}\left(\xi_{2}-\xi_{3}\right){\left(\xi_{2}-\xi_{3}\right)}^{{}^{\prime }}\Delta^{{}^{\prime }})+\text{tr}(\Delta \left(\xi_{1}\xi_{1}^{{}^{\prime }}+\Sigma \right)\left(\xi_{2}-\xi_{3}\right){\left(\varepsilon_{2}-\varepsilon_{3}\right)}^{{}^{\prime }})\\ & +\text{tr}{\left(\varepsilon_{1}\xi_{1}^{{}^{\prime }}\left(\xi_{2}-\xi_{3}\right)\left(\varepsilon_{2}-\varepsilon_{3}\right)\right)}^{{}^{\prime }}\}. \end{array} \end{array}$$

Based on the expansion of *X_i_*(*t*) and the orthogonality of the bases, we can derive the variance of the projections *v*_*c*_.

With straightforward calculations, we get

$$\begin{array}{ccc} v_{1} & = & \frac{1}{4}\{\text{tr}\left(M_{1}M_{3}\right)+\text{tr}\left(M_{2}^{2}\right)+\rho \text{tr}\left(Q_{2}\circ Q_{2}\right)+\text{tr}\left(\Lambda M_{3}\right)\},\\ v_{2} & = & \frac{1}{36}\{15\text{tr}\left(M_{2}^{2}\right)+28{\text{tr}}^{2}\left(M_{2}\right)+17\text{tr}\left(M_{1}M_{3}\right)+18\text{tr}\left(\Lambda M_{3}\right)+\text{tr}\left(M_{1}^{2}\right)\text{tr}\left(\Sigma^{2}\right)\\ & & +2\text{tr}\left(\Lambda M_{1}\right)\text{tr}\left(\Sigma^{2}\right)+15\rho \text{tr}\left(Q_{2}\circ Q_{2}\right)+\rho^{2}\text{tr}{\left(Q_{0}\circ Q_{1}\right)}^{2}\\ & & +2\rho \text{tr}\left(\Gamma^{{}^{\prime }}\Delta^{{}^{\prime }}\Lambda \Delta \Gamma \circ Q_{3}\right)+2\rho \text{tr}\left(Q_{3}\circ Q_{4}\right)+\text{tr}\left(\Lambda^{2}\right)\text{tr}\left(\Sigma^{2}\right)\}-{\text{tr}}^{2}\left(M_{2}\right),\\ v_{4} & = & \frac{1}{48}\{\left(62+12\rho \right)\text{tr}\left(M_{2}^{2}\right)+\left(70+8\rho \right){\text{tr}}^{2}\left(M_{2}\right)+130\text{tr}\left(M_{1}M_{3}\right)\\ & & +\left(46\rho +2\right)\text{tr}\left(Q_{2}\circ Q_{2}\right)+\left(34+4\rho \right)\text{tr}\left(M_{1}^{2}\right)\text{tr}\left(\Sigma^{2}\right)+14\rho \text{tr}\left(Q_{3}\circ Q_{4}\right)\\ & & +128\text{tr}\left(\Lambda M_{3}\right)+48\text{tr}\left(\Lambda M_{1}\right)\text{tr}\left(\Sigma^{2}\right)+24\rho \text{tr}\left(\Gamma^{{}^{\prime }}\Delta^{{}^{\prime }}\Lambda \Delta \Gamma \circ Q_{3}\right)\\ & & +8\rho^{2}\text{tr}{\left(Q_{0}\circ Q_{1}\right)}^{2}+24\text{tr}\left(\Lambda^{2}\right)\text{tr}\left(\Sigma^{2}\right)\}-{\text{tr}}^{2}\left(M_{2}\right). \end{array}$$

Here, the Hadamard product is defined as *A* ∘ *B* = (*a_ij_b_ij_*) for matrices *A* = (*a_ij_*) and *B* = (*b_ij_*). Since both variances *v*_2_ and *v*_4_ are linear combinations of $\text{tr}(M_{2}^{2})$, tr^2^(*M*_2_), tr(*M*_1_ *M*_3_), tr(Λ*M*_3_), $\text{tr}(M_{1}^{2})$, tr(Λ*M*_1_)tr(Σ^2^), tr(*Q*_2_ ∘ *Q*_2_), tr(*Q*_0_ ∘ *Q*_1_)^2^, tr(Γ′Δ′ΛΔΓ ∘ *Q*_3_), tr(*Q*_3_ ∘ *Q*_4_), and tr(Σ^2^), they are of the same asymptotic order. This means that the statistic *T*_*n*_ is dominated by the first two terms corresponding to *V*_*n*1_ and *V*_*n*2_. Hence we can get the Hoeffding decomposition (11) of *T*_*n*_, $T_{n}-\theta \left(F\right)=\sum_{c=1}^{2}\left(\begin{array}{c} 4\\ c \end{array}\right){\left(\begin{array}{c} n\\ c \end{array}\right)}^{-1}\{V_{nc}+o_{p}\left(1\right)\},$ and $var\left[T_{n}\right]=\left\{\frac{16}{n}v_{1}+\frac{72}{n(n-1)}v_{2}\right\}\left\{1+o\left(1\right)\right\}.$ Then we complete the proof.

**Proof of Theorem** 2.

Using the inequalities in Lemma 3, under either the null hypothesis or the local alternative, we have

$$\begin{array}{ccc} v_{1} & \leq & \frac{1}{4}\{\text{tr}\left(M_{1}\right)\text{tr}\left(M_{3}\right)+\text{tr}\left(M_{1}\right)\text{tr}\left(M_{3}\right)+\rho \text{tr}\left(M_{1}\right)\text{tr}\left(M_{3}\right)+\text{tr}\left(\Lambda \right)\text{tr}\left(M_{3}\right)\}\\ & = & \{(\frac{1}{2}+\frac{1}{4}\rho )\text{tr}\left(M_{1}\right)+\text{tr}\left(\Lambda \right)\}\text{tr}\left(M_{3}\right)\\ & = & \left\{\left(\frac{1}{2}+\frac{1}{4}\rho \right)o\left(1\right)+\text{tr}\left(\Lambda \right)\}o(n^{-1}\text{tr}\left(\Sigma^{2}\right)\right)\\ & = & o(n^{-1}\text{tr}\left(\Sigma^{2}\right)\text{tr}\left(\Lambda \right)). \end{array}$$ (19)

$$\begin{array}{ccc} v_{2} & \leq & \frac{1}{36}\{15{\text{tr}}^{2}\left(M_{2}\right)+28{\text{tr}}^{2}\left(M_{2}\right)-36{\text{tr}}^{2}\left(M_{2}\right)+17\text{tr}\left(M_{1}M_{3}\right)\\ & & +18\text{tr}\left(\Lambda \right)\text{tr}\left(M_{3}\right)+{\text{tr}}^{2}\left(M_{1}\right)\text{tr}\left(\Sigma^{2}\right)+2\text{tr}\left(\Lambda \right)\text{tr}\left(M_{1}\right)\text{tr}\left(\Sigma^{2}\right)\\ & & +15\rho \text{tr}\left(M_{1}\right)\text{tr}\left(M_{3}\right)+\rho^{2}\text{tr}\left(\Sigma^{2}\right){\text{tr}}^{2}\left(M_{1}\right)+2\text{tr}\left(M_{3}\right)\text{tr}\left(M_{1}\right)\text{tr}\left(\Lambda \right)\\ & & +2\rho \text{tr}\left(\Sigma^{2}\right){\text{tr}}^{2}\left(M_{1}\right)+\text{tr}\left(\Lambda^{2}\right)\text{tr}\left(\Sigma^{2}\right)\}\\ & \leq & \frac{1}{36}\{(24\text{tr}\left(M_{1}\right)+18\text{tr}\left(\Lambda \right)+15\rho \text{tr}\left(M_{1}\right)+2\text{tr}\left(M_{1}\right)\text{tr}\left(\Lambda \right))\text{tr}\left(M_{3}\right)\\ & & +({\text{tr}}^{2}\left(M_{1}\right)+2\text{tr}\left(\Lambda \right)\text{tr}\left(M_{1}\right)+\rho^{2}{\text{tr}}^{2}\left(M_{1}\right)\\ & & +2\rho {\text{tr}}^{2}\left(M_{1}\right)+\text{tr}\left(\Lambda^{2}\right))\text{tr}\left(\Sigma^{2}\right)\}\\ & = & o(\frac{1}{n}\text{tr}\left(\Sigma^{2}\right)\text{tr}\left(\Lambda \right))+O(\text{tr}\left(\Lambda^{2}\right)\text{tr}\left(\Sigma^{2}\right))\\ & = & O(\text{tr}\left(\Lambda^{2}\right)\text{tr}\left(\Sigma^{2}\right)). \end{array}$$ (20)

Thus *v*_1_ = *o*(*n*^−1^ *v*_2_).

Define

$$\begin{array}{c} {\hat{T}}_{n}={\left\|C\left(\delta_{\beta }\right)\right\|}^{2}+\frac{12}{n(n-1)}\sum_{1\leq i_{1}<i_{2}\leq n}{\tilde{\psi }}_{2}\left(w_{i_{1}},w_{i_{2}}\right), \end{array}$$ (21)

where *δ*_*β*_ = *β* − *β*_0_. Then we can get $T_{n}-\|C\delta_{\beta }{\|}^{2}=\left(T_{n}-{\hat{T}}_{n}\right)+({\hat{T}}_{n}-\|C\delta_{\beta }{\|}^{2}).$ We have

$$\begin{array}{c} T_{n}-{\hat{T}}_{n}={\left(\begin{array}{c} n\\ 4 \end{array}\right)}^{-1}\sum_{A}\{{\tilde{\psi }}_{4}\left(w_{i_{1}},w_{i_{2}},w_{i_{3}},w_{i_{4}}\right)-\sum_{1\leq k_{1}<k_{2}\leq 4}{\tilde{\psi }}_{2}\left(w_{i_{k_{1}}},w_{i_{k_{2}}}\right)\}, \end{array}$$

which can be regarded as a U-statistic with the kernel

$$\begin{array}{c} \Psi \left(w_{1},w_{2},w_{3},w_{4}\right)={\tilde{\psi }}_{4}\left(w_{1},w_{2},w_{3},w_{4}\right)-\sum_{1\leq i_{1}<i_{2}\leq 4}{\tilde{\psi }}_{2}\left(w_{i_{1}},w_{i_{2}}\right). \end{array}$$

Through direct calculation, we can get the projections of Ψ, $\Psi_{1}\left(w_{1}\right)=-2{\tilde{\psi }}_{1}\left(w_{1}\right)$, $\Psi_{2}\left(w_{1}.w_{2}\right)=-2\sum_{i=1}^{2}{\tilde{\psi }}_{1}\left(w_{i}\right)$, and $\Psi_{3}={\tilde{\psi }}_{3}\left(w_{1},w_{2},w_{3}\right)-\sum_{i=1}^{3}{\tilde{\psi }}_{1}\left(w_{i}\right)-\sum_{1\leq i<j\leq 3}{\tilde{\psi }}_{2}\left(w_{i},w_{j}\right)$. By Hoeffding’s variance decomposition, we have $\text{var}\left[{\hat{T}}_{n}\right]=O\left(n^{-2}v_{2}\right)$, $\text{var}\left[T_{n}-{\hat{T}}_{n}\right]=o\left(n^{-2}v_{2}\right)$ (see Supplementary Material B in S1 File).

Because

$$\begin{array}{c} \frac{T_{n}-{\left\|C\delta_{\beta }\right\|}^{2}}{\sqrt{\text{var}({\hat{T}}_{n})}}=\frac{T_{n}-{\hat{T}}_{n}}{\sqrt{\text{var}({\hat{T}}_{n})}}+\frac{{\hat{T}}_{n}-{\left\|C\delta_{\beta }\right\|}^{2}}{\sqrt{\text{var}({\hat{T}}_{n})}}=\frac{{\hat{T}}_{n}-{\left\|C\delta_{\beta }\right\|}^{2}}{\sqrt{\text{var}({\hat{T}}_{n})}}+o_{p}\left(1\right), \end{array}$$

we only need to show that

$$\begin{array}{c} \frac{{\hat{T}}_{n}-{\left\|C\delta_{\beta }\right\|}^{2}}{\sqrt{\text{var}({\hat{T}}_{n})}}\overset{P}{\to }N\left(0,1\right). \end{array}$$

From Eq (21) and the form of ${\tilde{\psi }}_{2}$, let ${\hat{T}}_{n}-{\left\|C\delta_{\beta }\right\|}^{2}={\hat{T}}_{n}^{\left(1\right)}+{\hat{T}}_{n}^{\left(2\right)}$, where

$$\begin{array}{ccc} {\hat{T}}_{n}^{\left(1\right)} & = & {\left(\begin{array}{c} n\\ 2 \end{array}\right)}^{-1}\sum_{1\leq i_{1}<i_{2}\leq n}\{\text{tr}(\Delta \left(\xi_{i_{1}}-\xi_{i_{2}}\right){\left(\xi_{i_{1}}-\xi_{i_{2}}\right)}^{{}^{\prime }}\Sigma \Delta^{{}^{\prime }})\\ & & +\text{tr}(\left(\varepsilon_{i_{1}}-\varepsilon_{i_{2}}\right){\left(\xi_{i_{1}}-\xi_{i_{2}}\right)}^{{}^{\prime }}\Sigma \Delta^{{}^{\prime }})+\text{tr}(\Delta \left(\xi_{i_{1}}\xi_{i_{1}}^{{}^{\prime }}+\Sigma \right)\left(\xi_{i_{2}}\xi_{i_{2}}^{{}^{\prime }}+\Sigma \right)\Delta^{{}^{\prime }})\\ & & +\text{tr}(\varepsilon_{i_{1}}\xi_{i_{1}}^{{}^{\prime }}\left(\xi_{i_{2}}\xi_{i_{2}}^{{}^{\prime }}+\Sigma \right)\Delta^{{}^{\prime }})+\text{tr}(\Delta \left(\xi_{i_{1}}\xi_{i_{1}}^{{}^{\prime }}+\Sigma \right)\xi_{i_{2}}\varepsilon_{i_{2}}^{{}^{\prime }})\}, \end{array}$$

and ${\hat{T}}_{n}^{\left(2\right)}={\left(\begin{array}{c} n\\ 2 \end{array}\right)}^{-1}\sum_{1\leq i_{1}<i_{2}\leq n}\text{tr}\left(\varepsilon_{i_{1}}\xi_{i_{1}}^{{}^{\prime }}\xi_{i_{2}}\varepsilon_{i_{2}}^{{}^{\prime }}\right).$ Under the assumptions of this theorem and following Eqs (19) and (20) we have $\text{var}\left[{\hat{T}}_{n}\right]=\text{var}\left[{\hat{T}}_{n}^{\left(2\right)}\right](1+o(1))$ and ${\hat{T}}_{n}^{\left(1\right)}/\sqrt{\text{var}({\hat{T}}_{n})}=o_{p}\left(1\right).$

To complete the proof, we now need to show

$$\begin{array}{c} {\hat{T}}_{n}^{\left(2\right)}/\sqrt{\text{var}({\hat{T}}_{n}^{\left(2\right)}})=\sqrt{n2}{\hat{T}}_{n}^{\left(2\right)}/\sqrt{\text{tr}\left(\Lambda^{2}\right)\text{tr}\left(\Sigma^{2}\right)}\overset{P}{\to }N\left(0,1\right). \end{array}$$

Define $Z_{ni}=\sum_{j=1}^{i-1}\varepsilon_{i}^{{}^{\prime }}\varepsilon_{j}\xi_{i}^{{}^{\prime }}\xi_{j}/\sqrt{n2}$ and ${\tilde{T}}_{nk}=\sum_{i=2}^{k}Z_{ni}$, thus ${\tilde{T}}_{nn}=\sqrt{n2}{\hat{T}}_{nk}^{\left(2\right)}$, which we define as ${\tilde{T}}_{n}$. Let $F_{i}=\sigma \left\{\left(\begin{array}{c} \xi_{1}\\ \varepsilon_{1} \end{array}\right),\ldots ,\left(\begin{array}{c} \xi_{i}\\ \varepsilon_{i} \end{array}\right)\right\}$ be a *σ*−field generated by $\left\{\left(\xi_{j}^{\prime },\varepsilon_{j}^{\prime }\right),j\leq i\right\}$. It is obvious to see that $\text{E}[Z_{ni}|F_{i-1}]=0$, $F_{1}\subset F_{2}\subset \ldots$. Then it shows that $\left\{{\tilde{T}}_{nk},F_{k}:2\leq k\leq n\right\}$ is a zero mean martingale. Let $V_{ni}^{*}=\text{E}\left[Z_{ni}^{2}|F_{i-1}\right],2\leq i\leq n$, $V_{n}^{*}=\sum_{i=2}^{n}V_{ni}^{*}$. The central limit theorem will hold Hall 28 if we can show $V_{n}^{*}$ satisfies the following two conditions:

$$\begin{array}{c} \frac{V_{n}^{*}}{\text{var}({\tilde{T}}_{n})}\overset{P}{\to }1, \end{array}$$ (22)

and for ∀*τ* > 0

$$\begin{array}{c} \sum_{i=1}^{n}{\text{tr}}^{-1}\left(\Lambda^{2}\right){\text{tr}}^{-1}\left(\Sigma^{2}\right)\text{E}\{Z_{ni}^{2}I(|Z_{ni}|>\tau \sqrt{\text{tr}\left(\Lambda^{2}\right)\text{tr}\left(\Sigma^{2}\right)})|F_{i-1}\}\overset{P}{\to }0. \end{array}$$

We have $\text{var}\left[{\tilde{T}}_{n}\right]=\text{tr}\left(\Lambda^{2}\right)\text{tr}\left(\Sigma^{2}\right)$ and

$$\begin{array}{c} V_{ni}^{*}={\left(\begin{array}{c} n\\ 2 \end{array}\right)}^{-1}(\sum_{j=1}^{i-1}\varepsilon_{j}^{{}^{\prime }}\Lambda \varepsilon_{j}\xi_{j}^{{}^{\prime }}\Sigma \xi_{j}+2\sum_{1\leq j<\ell \leq i}\varepsilon_{j}^{{}^{\prime }}\Lambda \varepsilon_{\ell }\xi_{j}^{{}^{\prime }}\Sigma \xi_{\ell }). \end{array}$$

Hence we can define $V_{n}^{*}/\text{var}\left({\tilde{T}}_{n}\right)=C_{n1}+C_{n2}$, where

$$\begin{array}{ccc} C_{n1} & = & \frac{1}{\left(\begin{array}{c} n\\ 2 \end{array}\right)\text{tr}\left(\Sigma^{2}\right)\text{tr}\left(\Lambda^{2}\right)}\sum_{j=1}^{n-1}j\varepsilon_{j}^{{}^{\prime }}\Lambda \varepsilon_{j}\xi_{j}^{{}^{\prime }}\Sigma \xi_{j},\\ C_{n2} & = & \frac{1}{\left(\begin{array}{c} n\\ 2 \end{array}\right)\text{tr}\left(\Sigma^{2}\right)\text{tr}\left(\Lambda^{2}\right)}2\sum_{1\leq j<\ell \leq i}\varepsilon_{j}^{{}^{\prime }}\Lambda \varepsilon_{\ell }\xi_{j}^{{}^{\prime }}\Sigma \xi_{\ell }. \end{array}$$

It can be shown that E[*C*_*n*1_] = 1, and

$$\begin{array}{c} \begin{array}{cc} \text{var}[C_{n1}]= & \frac{1}{{\left(\begin{array}{c} n\\ 2 \end{array}\right)}^{2}{\text{tr}}^{2}\left(\Sigma^{2}\right){\text{tr}}^{2}\left(\Lambda^{2}\right)}\{\text{E}[\sum_{j=1}^{n-1}j^{2}\varepsilon_{j}^{{}^{\prime }}\Lambda \varepsilon_{j}\varepsilon_{j}^{{}^{\prime }}\Lambda \varepsilon_{j}\xi_{j}^{{}^{\prime }}\Sigma \xi_{j}\xi_{j}^{{}^{\prime }}\Sigma \xi_{j}]\\ & -\sum_{j=1}^{n-1}j^{2}{\text{tr}}^{2}\left(\Sigma^{2}\right){\text{tr}}^{2}\left(\Lambda^{2}\right)\}. \end{array} \end{array}$$

As tr(Σ^4^) = *O*(tr^2^(Σ^2^)), and var[*C*_*n*1_ → 0. Then $C_{n1}\overset{P}{\to }1$ (see Supplementary Material B in S1 File). Similarly, E[*C*_*n*2_] = 0, $\text{var}\left[C_{n2}\right]=\frac{4}{{\left(\begin{array}{c} n\\ 2 \end{array}\right)}^{2}{\text{tr}}^{2}\left(\Sigma^{2}\right){\text{tr}}^{2}\left(\Lambda^{2}\right)}\text{tr}\left(\Lambda^{4}\right)\text{tr}\left(\Sigma^{4}\right)$, then $C_{n2}\overset{P}{\to }0.$ In summary, Eq (22) holds.

Since $\text{E}[Z_{ni}^{2}I\{|Z_{ni}|>\tau \sqrt{\text{tr}\left(\Lambda^{2}\right)\text{tr}\left(\Sigma^{2}\right)}\}|F_{i-1}]\leq \text{E}\left[Z_{ni}^{4}|F_{i-1}\right]/\left\{\tau^{2}\text{tr}\left(\Lambda^{2}\right)\text{tr}\left(\Sigma^{2}\right)\right\}$, by the law of large numbers, the last step is to prove

$$\begin{array}{c} \sum_{i=1}^{n}\text{E}\left[Z_{ni}^{4}\right]=o\left({\text{tr}}^{2}\left(\Lambda^{2}\right){\text{tr}}^{2}\left(\Sigma^{2}\right)\right). \end{array}$$ (23)

We have $\text{E}\left[Z_{ni}^{4}\right]={\left(\begin{array}{c} n\\ 2 \end{array}\right)}^{-2}\text{E}[\sum_{j=1}^{i-1}\left(i-2\right)\varepsilon_{j}^{{}^{\prime }}\Lambda \varepsilon_{j}\varepsilon_{j}^{{}^{\prime }}\Lambda \varepsilon_{j}\xi_{j}^{{}^{\prime }}\Sigma \xi_{j}\xi_{j}^{{}^{\prime }}\Sigma \xi_{j}+\sum_{j=1}^{i-1}{\left(\varepsilon_{i}^{{}^{\prime }}\varepsilon_{j}\right)}^{4}{\left(\xi_{i}^{{}^{\prime }}\xi_{j}\right)}^{4}]$, and $\text{E}{\left[\xi_{i}^{{}^{\prime }}\xi_{j}\right]}^{4}=6\text{tr}\left(\Sigma^{4}\right)+\left(6\rho +\rho^{2}+4\right)\text{tr}\left(Q_{3}\circ Q_{3}\right)+2{\text{tr}}^{2}\left(\Sigma^{2}\right)$, thus under Assumption (16), we have (see details in S1 File)

$$\begin{array}{ccc} \sum_{i=1}^{n}\text{E}\left[Z_{ni}^{4}\right] & \leq & {\left(\begin{array}{c} n\\ 2 \end{array}\right)}^{-2}\sum_{i=1}^{n}\{2\left(\begin{array}{c} i-1\\ 2 \end{array}\right)+(i-1)\}O({\text{tr}}^{2}\left(\Lambda^{2}\right){\text{tr}}^{2}\left(\Sigma^{2}\right))\\ & = & O(n^{-1}{\text{tr}}^{2}\left(\Lambda^{2}\right){\text{tr}}^{2}\left(\Sigma^{2}\right))\\ & = & o({\text{tr}}^{2}\left(\Lambda^{2}\right){\text{tr}}^{2}\left(\Sigma^{2}\right)). \end{array}$$

Hence we prove that Eq (23) holds. And this completes the proof.

## Supporting information

S1 File (PDF)
