# Supplementary material for: Nonparametric testing of lack of dependence in functional linear models
Source: PLoS One. 2020 Jun 26;15(6):e0234094. doi: 10.1371/journal.pone.0234094 (PMC7319281; doi:10.1371/journal.pone.0234094)
Supplement: S1 File — (PDF) [file pone.0234094.s001.pdf]

Supplementary Material A. Numerical instability of the NP method.

First we conduct a simulation study to evaluate the empirical size and power of the NP method under three different ways of searching the direction γ. The simulation setup is the same as that in Section Simulation results. Here we presents the results under the low dimension case ($K = L = 5$) and sample size $n = 40$. In Table 1, results in the two columns under "case 1" are based on estimating γ in the idea situation, i.e. pre-estimating it using a super large data set simulated under the null case. Results in the two columns under "case 2" are based on pre-estimating γ from a super large data set simulated at each level of $|\beta|^2$. And the last two columns under "case 3" use γ estimated based on each individual simulate data set.

In Table 1, results from the case 1 always utilize the same estimate of γ, which may not be correct for cases with $|\beta|^2 > 0$. So, we see that the nominal significance level is well respected at $|\beta|^2 = 0$. But the test power increased quite slowly as $|\beta|^2$ increases. For example, when $|\beta|^2 = 0.50$, the power reached only 0.463 at the 0.1 significance level. On the other hand, when γ is estimated according to the true setup of $|\beta|^2$, the test power is much improved under the column case 2. But when using the NP method, the most realistic situation is the case 3 situation because the only available information to estimate γ is the given data set. Unfortunately, the NP method displays severe numerical instability as the direction estimated based on a single simulated data set of size $n = 40$ can be highly variable. For example, test power at $|\beta|^2 = 0.30$ is only around 20%.

Supplementary Material B. Proof details.

This section contains some details for the proof of Theorem 2.

1.$\text{var}[\hat{T}_n] = O(n^{-2}v_2)$, and $\text{var}[T_n - \hat{T}_n] = o(n^{-2}v_2)$.

Proof. From Equations (19) and (20), we know that $v_1 = o(n^{-1}v_2)$, and use the

definition of $\hat{T}_n$, we have

$$
\begin{aligned}
\mathrm{var}[\hat{T}_n] &= \Big(\frac{12}{n(n-1)}\Big)^2 \mathrm{var}\Big[\sum_{1\le i_1<i_2\le n} \tilde{\psi}_2(w_{i_1}, w_{i_2})\Big] \\
&= \Big(\frac{12}{n(n-1)}\Big)^2 \mathrm{E}\Big[\sum_{1\le i_1<i_2\le n} \tilde{\psi}_2^2(w_{i_1}, w_{i_2}) \\
&\quad + \sum_{1\le i_1<i_2<i_3\le n} 3\tilde{\psi}_2^2(w_{i_1}, w_{i_2})\tilde{\psi}_2^2(w_{i_1}, w_{i_3}) \\
&\quad + \sum_{1\le i_1<i_2<i_3<i_4\le n} \tilde{\psi}_2^2(w_{i_1}, w_{i_2})\tilde{\psi}_2^2(w_{i_3}, w_{i_4})\Big] \\
&= \Big(\frac{12}{n(n-1)}\Big)^2 \Big(\sum_{1\le i_1<i_2\le n} v_2 + \sum_{1\le i_1<i_2<i_3\le n} 3v_1\Big) \\
&= O(n^{-2}v_2).
\end{aligned}
$$

Through calculating the variance of the projections $\Psi(w_{i_1}, w_{i_2}, w_{i_3}, w_{i_4})$, and $v_1 = o(n^{-1}v_2)$, we have

$$
\begin{aligned}
&\mathrm{var}[\mathrm{T}_n - \hat{T}_n] \\
&= \mathrm{var}\Big[\binom{n}{4}^{-1} \sum_{\mathcal{A}} \Psi(w_{i_1}, w_{i_2}, w_{i_3}, w_{i_4})\Big] \\
&= \binom{n}{4}^{-2} \sum_{\mathcal{A}_1}\sum_{\mathcal{A}_2} \mathrm{E}\Big[\Big\{\tilde{\psi}_4(w_{i_1}, w_{i_2}, w_{i_3}, w_{i_4}) - \sum_{1\le i_1<i_2\le n} \tilde{\psi}_2(w_{i_1}, w_{i_2})\Big\} \\
&\quad \Big\{\tilde{\psi}_4(w_{j_1}, w_{j_2}, w_{j_3}, w_{j_4}) - \sum_{1\le j_1<j_2\le n} \tilde{\psi}_2(w_{j_1}, w_{j_2})\Big\}\Big] \\
&= \binom{n}{4}^{-2} \sum_{\mathcal{A}} \mathrm{E}\Big[\Big\{\tilde{\psi}_4(w_{j_1}, w_{j_2}, w_{j_3}, w_{j_4}) - \sum_{1\le j_1<j_2\le n} \tilde{\psi}_2(w_{j_1}, w_{j_2})\Big\} \\
&\quad \Big\{\binom{4}{1}\binom{n-4}{3}\Big(\tilde{\psi}_4(w_{i_1}, w_{j_2}, w_{j_3}, w_{j_4}) - 3\tilde{\psi}_2(w_{i_1}, w_{j_2})\Big) \\
&\quad + \binom{4}{2}\binom{n-4}{2}\Big(\tilde{\psi}_4(w_{i_1}, w_{i_2}, w_{j_3}, w_{j_4}) - \tilde{\psi}_2(w_{i_1}, w_{i_2}) - 4\tilde{\psi}_2(w_{i_1}, w_{j_3})\Big) \\
&\quad + \binom{4}{3}\binom{n-4}{1}\Big(\tilde{\psi}_4(w_{i_1}, w_{i_2}, w_{i_3}, w_{j_4}) - 3\tilde{\psi}_2(w_{i_1}, w_{i_2}) - 3\tilde{\psi}_2(w_{i_1}, w_{j_4})\Big) \\
&\quad + \binom{4}{4}\Big(\tilde{\psi}_4(w_{i_1}, w_{i_2}, w_{i_3}, w_{i_4}) - 6\tilde{\psi}_2(w_{i_1}, w_{i_2})\Big)\Big\}\Big] \\
&= \binom{n}{4}^{-1}\Big\{16\binom{n-4}{3}v_1 + 48\binom{n-4}{2}v_1 + o(n^{-2}v_1)\Big\} \\
&= o(n^{-2}v_2).
\end{aligned}
$$

2. $C_{n1} \xrightarrow{P} 1$.

Proof. The expectation of C_{n1} is

$$
\mathrm{E}[C_{n1}] = \frac{1}{\binom{n}{2}\mathrm{tr}(\Sigma^2)\mathrm{tr}(\Lambda^2)} \sum_{j=1}^{n-1} E\big[j\boldsymbol{\varepsilon}_j^{'}\Lambda\boldsymbol{\varepsilon}_j\boldsymbol{\xi}_j^{'}\Sigma\boldsymbol{\xi}_j\big] = 1,
$$

and the variance of C_{n1} is

var[C$_{n1}$]

$$\begin{aligned}
&= \frac{1}{\binom{n}{2}^2 \mathrm{tr}^2(\Sigma^2)\mathrm{tr}^2(\Lambda^2)}\Big\{\mathrm{E}\Big[\Big(\sum_{j=1}^{n-1} j\boldsymbol{\varepsilon}_j^{'}\Lambda\boldsymbol{\varepsilon}_j\boldsymbol{\xi}_j^{'}\Sigma\boldsymbol{\xi}_j\Big)^2\Big] - \binom{n}{2}^2 \mathrm{tr}^2(\Sigma^2)\mathrm{tr}^2(\Lambda^2)\Big\} \\
&= \frac{1}{\binom{n}{2}^2 \mathrm{tr}^2(\Sigma^2)\mathrm{tr}^2(\Lambda^2)}\Big\{\mathrm{E}\Big[\sum_{i\neq j} ij\boldsymbol{\varepsilon}_i^{'}\Lambda\boldsymbol{\varepsilon}_i\boldsymbol{\varepsilon}_j^{'}\Lambda\boldsymbol{\varepsilon}_j\boldsymbol{\xi}_i^{'}\Sigma\boldsymbol{\xi}_i\boldsymbol{\xi}_j^{'}\Sigma\boldsymbol{\xi}_j + \sum_{j=1}^{n-1} j^2(\boldsymbol{\varepsilon}_j^{'}\Lambda\boldsymbol{\varepsilon}_j\boldsymbol{\xi}_j^{'}\Sigma\boldsymbol{\xi}_j)^2\Big] \\
&\quad - \binom{n}{2}^2 \mathrm{tr}^2(\Sigma^2)\mathrm{tr}^2(\Lambda^2)\Big\} \\
&= \frac{1}{\binom{n}{2}^2 \mathrm{tr}^2(\Sigma^2)\mathrm{tr}^2(\Lambda^2)}\sum_{j=1}^{n-1} j^2\Big\{\mathrm{E}\Big[\boldsymbol{\varepsilon}_j^{'}\Lambda\boldsymbol{\varepsilon}_j\boldsymbol{\varepsilon}_j^{'}\Lambda\boldsymbol{\varepsilon}_j\boldsymbol{\xi}_j^{'}\Sigma\boldsymbol{\xi}_j\boldsymbol{\xi}_j^{'}\Sigma\boldsymbol{\xi}_j\Big] - \mathrm{tr}^2(\Sigma^2)\mathrm{tr}^2(\Lambda^2)\Big\}.
\end{aligned}$$

Under Assumption 2, $\mathrm{E}[(\boldsymbol{\varepsilon}_i^{'}\Lambda\boldsymbol{\varepsilon}_i)^2] = O(\mathrm{tr}^2(\Lambda^2))$. And from Lemma 3 , we have $\mathrm{tr}(\Sigma^4) = O(\mathrm{tr}^2(\Sigma^2))$, thus we show that

$$\begin{aligned}
\mathrm{E}\Big[\boldsymbol{\xi}_j^{'}\Sigma\boldsymbol{\xi}_j\boldsymbol{\xi}_j^{'}\Sigma\boldsymbol{\xi}_j\Big] &= 2\mathrm{tr}(\Sigma^4) + \rho\mathrm{tr}(Q_3 \circ Q_3) + \mathrm{tr}^2(\Sigma^2) \\
&\leq (1+\rho)\mathrm{tr}^2(\Sigma^2)\{1 + o_p(1)\}.
\end{aligned}$$

Hence $\mathrm{var}[C_{n1}] \to 0$, then $C_{n1} \xrightarrow{P} 1$.

3. $\sum_{i=1}^n \mathrm{E}[Z_{ni}^4] = o(\mathrm{tr}^2(\Lambda^2)\mathrm{tr}^2(\Sigma^2))$.

Proof. We have

$\mathrm{E}[Z_{ni}^4] = \binom{n}{2}^{-2}\mathrm{E}\Big[\sum_{j=1}^{i-1}(i-2)\boldsymbol{\varepsilon}_j^{'}\Lambda\boldsymbol{\varepsilon}_j\boldsymbol{\varepsilon}_j^{'}\Lambda\boldsymbol{\varepsilon}_j\boldsymbol{\xi}_j^{'}\Sigma\boldsymbol{\xi}_j\boldsymbol{\xi}_j^{'}\Sigma\boldsymbol{\xi}_j + \sum_{j=1}^{i-1}(\boldsymbol{\varepsilon}_i^{'}\boldsymbol{\varepsilon}_j)^4(\boldsymbol{\xi}_i^{'}\boldsymbol{\xi}_j)^4\Big]$, from Lemma 7.1 and $\mathrm{tr}(\Sigma^4) = O(\mathrm{tr}^2(\Sigma^2))$, thus

$$\begin{aligned}
\mathrm{E}[\boldsymbol{\xi}_j^{'}\Sigma\boldsymbol{\xi}_j\boldsymbol{\xi}_j^{'}\Sigma\boldsymbol{\xi}_j] &= 2\mathrm{tr}(\Sigma^4) + \mathrm{tr}^2(\Sigma^2) + \rho\mathrm{tr}(Q_3 \circ Q_3) \\
&\leq 2\mathrm{tr}^2(\Sigma^2) + \mathrm{tr}^2(\Sigma^2) + \rho\mathrm{tr}^2(\Sigma^2) \\
&= O(\mathrm{tr}^2(\Sigma^2)),
\end{aligned}$$

and

$$\begin{aligned}
\mathrm{E}[\boldsymbol{\xi}_i^{'}\boldsymbol{\xi}_j]^4 &= 6\mathrm{tr}(\Sigma^4) + (6\rho + \rho^2 + 4)\mathrm{tr}(Q_3 \circ Q_3) + 2\mathrm{tr}^2(\Sigma^2) \\
&\leq 6\mathrm{tr}^2(\Sigma^2) + (6\rho + \rho^2 + 4)\mathrm{tr}^2(\Sigma^2) + 2\mathrm{tr}^2(\Sigma^2) \\
&= O(\mathrm{tr}^2(\Sigma^2)).
\end{aligned}$$

From Assumption 2, we have

$$\mathrm{E}[\boldsymbol{\varepsilon}_j^{'}\Lambda\boldsymbol{\varepsilon}_j\boldsymbol{\varepsilon}_j^{'}\Lambda\boldsymbol{\varepsilon}_j] = O(\mathrm{tr}^2(\Lambda^2)) \quad \text{and} \quad \mathrm{E}[(\boldsymbol{\varepsilon}_i^{'}\boldsymbol{\varepsilon}_j)^4] = O(\mathrm{tr}^2(\Lambda^2)).$$

Then

$$\begin{aligned}
\sum_{i=1}^n \mathrm{E}[Z_{ni}^4] &\leq \binom{n}{2}^{-2}\sum_{i=1}^n\Big\{2\binom{i-1}{2} + (i-1)\Big\}O\big(\mathrm{tr}^2(\Lambda^2)\mathrm{tr}^2(\Sigma^2)\big) \\
&= o(\mathrm{tr}^2(\Lambda^2)\mathrm{tr}^2(\Sigma^2).
\end{aligned}$$
